# Chloroplast envelope ATPase PGA1/AtFtsH12 is required for chloroplast protein accumulation and cytosol-chloroplast protein homeostasis in *Arabidopsis*

**DOI:** 10.1016/j.jbc.2022.102489

**Published:** 2022-09-14

**Authors:** Qinglong Li, Xiaomin Wang, Yang Lei, Yanling Wang, Bilang Li, Xiayan Liu, Lijun An, Fei Yu, Yafei Qi

**Affiliations:** State Key Laboratory of Crop Stress Biology for Arid Areas and College of Life Sciences, Northwest A&F University, Yangling, Shaanxi, China

**Keywords:** AtFtsH12, chloroplast protein accumulation, photosynthetic complexes, cytosol-chloroplast protein homeostasis, cpUPR, AAA, ATPases associated with diverse cellular activities, BN-PAGE, blue native PAGE, cpUPR, chloroplast-unfolded protein response, LHC, light-harvesting antenna complex, NEP, nucleus-encoded RNA polymerase, PEP, plastid-encoded RNA polymerase, PSI, Photosystem I, PSII, Photosystem II, RuBisCO, ribulose-1,5-bisphosphate carboxylase/oxygenase

## Abstract

The establishment of photosynthetic protein complexes during chloroplast development requires the influx of a large number of chloroplast proteins that are encoded by the nuclear genome, which is critical for cytosol and chloroplast protein homeostasis and chloroplast development. However, the mechanisms regulating this process are still not well understood in higher plants. Here, we report the isolation and characterization of the *pale green Arabidopsis pga1-1* mutant, which is defective in chloroplast development and chloroplast protein accumulation. Using genetic and biochemical evidence, we reveal that *PGA1* encodes AtFtsH12, a chloroplast envelope-localized protein of the FtsH family proteins. We determined a G703R mutation in the GAD motif of the conserved ATPase domain renders the *pga1-1* a viable hypomorphic allele of the essential gene *AtFtsH12*. In de-etiolation assays, we showed that the accumulation of photosynthetic proteins and the expression of photosynthetic genes were impaired in *pga1-1*. Using the FNR_ctp_-GFP and pTAC2-GFP reporters, we demonstrated that AtFtsH12 was required for the accumulation of chloroplast proteins *in vivo*. Interestingly, we identified an increase in expression of the mutant *AtFtsH12* gene in *pga1-1*, suggesting a feedback regulation. Moreover, we found that cytosolic and chloroplast proteostasis responses were triggered in *pga1-1*. Together, taking advantage of the novel *pga1-1* mutant, we demonstrate the function of AtFtsH12 in chloroplast protein homeostasis and chloroplast development.

In the chloroplast, the physical and chemical processes of photochemistry and carbon assimilation are mediated by multiple photosynthetic protein complexes such as Photosystem II (PSII), Photosystem I (PSI), Cytochrome b_6_f, and ATP synthase for light reactions, and ribulose-1,5-bisphosphate carboxylase/oxygenase (RuBisCO) for carbon reactions. As a consequence of endosymbiosis, many photosynthetic protein complexes are composed of subunits encoded by both photosynthesis-associated nuclear genes (*PhANGs*) and genes in the chloroplast genome (1). During chloroplast development, the expression of many *PhANGs* is activated by light signaling (2). *PhANG* transcripts are translated in the cytosol and their protein products are subsequently translocated into the chloroplast through the TOC/TIC system (translocon at the outer/inner envelope membrane of chloroplasts) (3). In the chloroplast, photosynthetic genes in the chloroplast genome are predominantly transcribed by the plastid-encoded RNA polymerase (PEP), and the *rpo* genes encoding PEP core subunits are transcribed by the nucleus-encoded RNA polymerase (NEP) (4). Thus, the establishment of functional photosynthetic protein complexes during chloroplast development is a critically important and highly complex process, requiring the coordination of both anterograde signaling from the nucleus to control chloroplast gene expression and retrograde signaling from the chloroplast to regulate nuclear gene expression (1, 2, 5, 6, 7, 8).

The chimeric composition of photosynthetic protein complexes poses an enormous challenge for maintaining protein homeostasis (proteostasis) and numerous chaperone and protease systems are involved in the regulation of proteostasis in the cytosol and in the chloroplast (6, 9, 10). In the cytosol, the disruption of chloroplast gene expression or protein import leads to cytosolic protein stress (11, 12). In vascular plants, the heat shock transcription factors are involved in the cytosol protein-stress response, similar to heat shock responses in yeast and mammalian cells (13, 14). In the chloroplast, protein quality responses were observed in WT Arabidopsis treated with lincomycin to block chloroplast translation (15) or in the *var2* mutant with impaired photosystem II repair cycle (16). Numerous chaperones and proteases are also present in the chloroplast (10). For example, stroma chaperones such as HSP90 and HSP70 are important components of the protein quality control systems (17). In addition, the serine protease Clp is the main proteolytic machinery in the chloroplast stroma (18). In Chlamydomonas, the conditional depletion of chloroplast ClpP1 induced the expression of nuclear genes involved in plastid protein quality control such as chloroplast HSP70 and an involvement of ClpP1 in chloroplast-unfolded protein stress response was proposed (19, 20).

In eukaryotes, FtsH family of ATPases associated with diverse cellular activities (AAA)+ proteins plays key roles in protein quality control in different organelles (21). In Arabidopsis, AtFtsH3, AtFtsH4, and AtFtsH10 are targeted into mitochondria and are involved in the assembly or stability of the oxidative phosphorylation complexes (22, 23, 24). The thylakoid FtsH complexes, consisting of AtFtsH1, VAR1/AtFtsH5, VAR2/AtFtsH2, and AtFtsH8, are heterohexamers involved in the PSII repair cycle and chloroplast development (22, 25, 26, 27, 28, 29). In the chloroplast envelope, AtFtsH12 forms the heteromeric AtFtsH12–FtsHi (FtsH inactive) complexes with several members of the FtsHi protein family and interacts with the TIC complex and the plastid NAD-dependent Malate Dehydrogenases (30, 31, 32). Null mutations of components of the AtFtsH12–FtsHi complexes, such as AtFtsH12, FtsHi1, FtsHi2, FtsHi4, FtsHi5, or plastid NAD-dependent Malate Dehydrogenase, give rise to embryonic lethal phenotypes, indicating the essential nature of these proteins (3, 30, 31, 33). The ATPase activity of the AtFtsH12–FtsHi complex was proposed to provide the driving force for chloroplast protein translocations (30). This is similar to the yeast mitochondrial FtsH homolog YME1, which uses the energy of ATP hydrolysis to drive the translocation of substrates into the proteolytic domain (34).

In this work, we report a chloroplast development mutant *pale green Arabidopsis 1-1* (*pga1-1*), which showed defects in the accumulation of photosynthetic proteins and photosynthetic gene expression. Map-based cloning and molecular complementation confirmed that *PGA1* encodes AtFtsH12. Biochemical evidence showed that AtFtsH12 is a part of large complexes. Furthermore, AtFtsH12 is required for the accumulation of photosynthetic proteins and photosynthetic gene expression during de-etiolation. Moreover, the defective accumulation of chloroplast proteins in *pga1-1* induced cytosolic and chloroplast proteostasis responses. Our results demonstrate that AtFtsH12 plays important roles in chloroplast protein accumulation and cytosol-chloroplast protein homeostasis.

## Results

### The identification of the *pale green Arabidopsis 1-1* mutant

**Figure 1:**
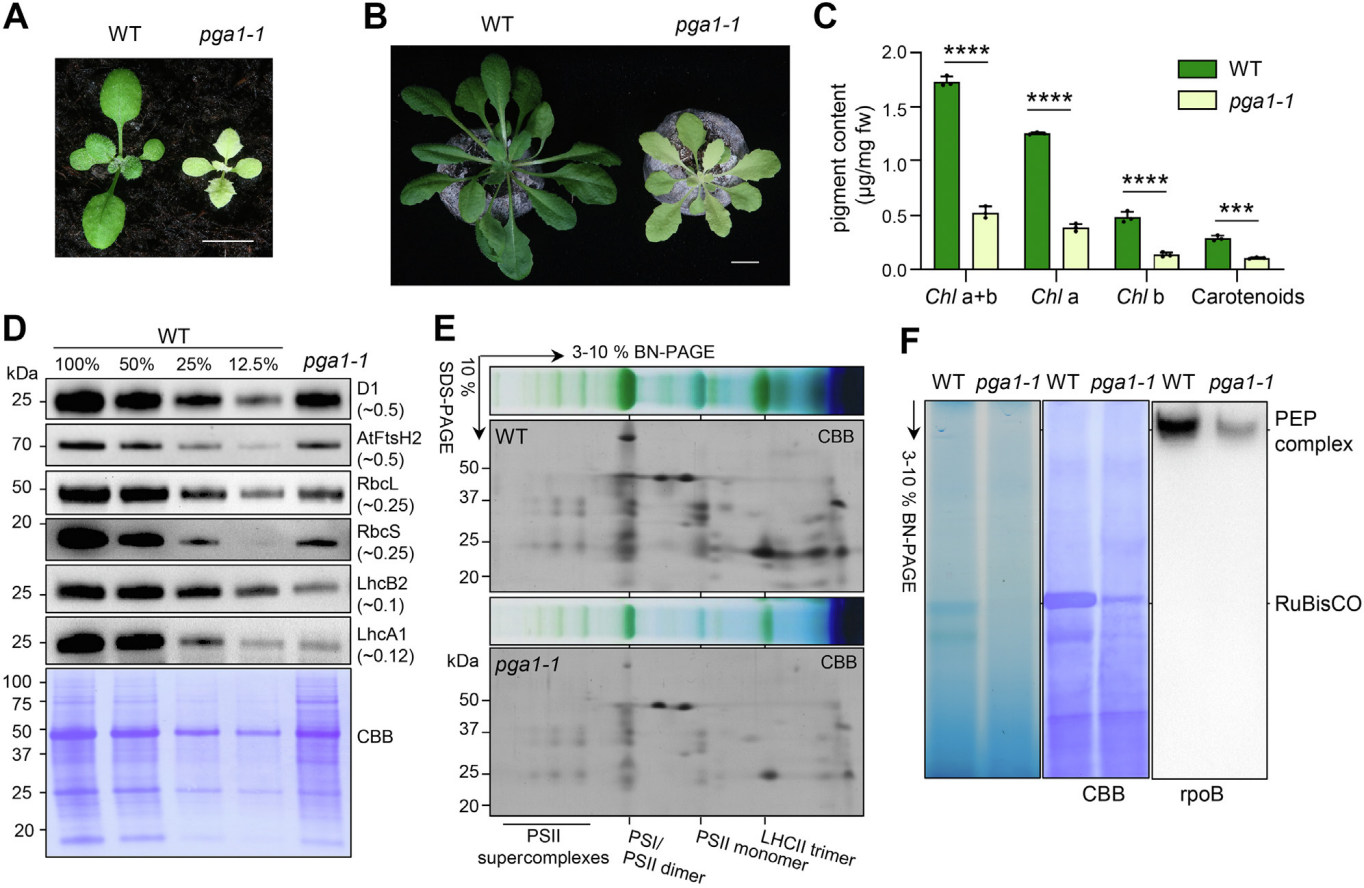
**Phenotypes of the *pga1-1* mutant.***A*, representative 2-week-old seedlings of WT and *pga1-1* grown under continuous light. The scale bar represents 0.5 cm. *B*, representative 40-day-old WT and *pga1-1* grown under a day/night cycle (12/12 h). The scale bar represents 1 cm. *C*, the accumulation of chlorophylls and carotenoids in 2-week-old WT and *pga1-1*. ∗∗∗0.0001 < *p* < 0.001, ∗∗∗∗*p* < 0.0001. *D*, the steady-state levels of photosynthetic proteins in 2-week-old WT and *pga1-1*. The relative levels of proteins in *pga1-1* compared with those in the WT were calculated based on the immunoblotting signal intensities and shown in the parentheses. Protein loading was normalized to equal fresh tissue weight and confirmed by Coomassie Brilliant Blue (CBB)-stained PVDF membranes. *E*, the accumulation of photosynthetic complexes in WT and *pga1-1* was analyzed with 1-D BN-PAGE and 2-D SDS-PAGE. Thylakoids were solubilized with 1% β-DM. The 1-D BN-PAGE gel lanes for 2-D SDS-PAGE were those shown in Fig. S2*A*. *F*, the accumulation of RuBisCO (indicated by CBB staining) and PEP complexes (indicated by immunoblotting the 1-D BN-PAGE using an anti-rpoB monoclonal antibody) in 4-day-old seedlings. Protein loading was normalized to equal fresh tissue weight and confirmed by CBB-stained PVDF membranes. β-DM, 1% n-dodecyl-β-D-maltoside; BN-PAGE, blue native PAGE; RuBisCO, ribulose-1,5-bisphosphate carboxylase/oxygenase; PEP, plastid-encoded RNA polymerase.

To dissect the molecular mechanisms underpinning chloroplast development, we have systematically isolated and characterized different categories of leaf color mutants in Arabidopsis, including *virescent* (35), *variegation* (36), and *pale green Arabidopsis* (*pga*) mutants. The *pga1-1* mutant showed a characteristic pale-green leaf color phenotype in both juvenile and adult stages, and a reduced plant stature, compared with the WT (Figs. 1, *A* and *B* and S1). Consistent with the pale-green appearance, the chlorophyll content in rosette leaves of 2-week-old *pga1-1* was reduced to ∼30% of that in the WT (Fig. 1*C*). Next, we checked the steady-state accumulation of photosynthetic proteins in 2-week-old *pga1-1* plants (Fig. 1*D*). The PSII reaction center subunit D1 and the thylakoid FtsH complex subunit VAR2/AtFtsH2 were reduced to about 50% of those in the WT, while RbcL and RbcS, the large and small subunits of RuBisCO, respectively, accumulated to ∼25% of those in the WT (Fig. 1*D*). Interestingly, the levels of light-harvesting antenna complex (LHC) subunits, such as LhcB2 for PSII and LhcA1 for PSI, showed the strongest reductions in *pga1-1*, to only ∼10% of those in the WT (Fig. 1*D*).

Next, we analyzed the accumulation of photosynthetic protein complexes with blue native PAGE (BN-PAGE). Thylakoid membranes isolated from WT and *pga1-1* were solubilized with 1% n-dodecyl-β-D-maltoside or 2% digitonin (Fig. S2, *A* and *B*). Overall, 1-D BN-PAGE revealed that major complexes, such as PSII supercomplexes, PSI, PSII dimers, and monomers, as well as LHCII trimers, still accumulated in *pga1-1*, albeit to significantly reduced levels compared with the WT (Fig. S2, *A* and *B*), and 2-D denaturing BN-PAGE further confirmed the general reduction of photosynthetic protein complexes and subunits (Figs. 1*E* and S2*C*). In addition, soluble proteins from 4-day-old WT and *pga1-1* seedlings were resolved on 1-D BN-PAGE, and the accumulations of RuBisCO and PEP complexes were also significantly reduced in *pga1-1* (Fig. 1*F*).

Collectively, these data establish that *PGA1* is required for chloroplast development and the accumulation of photosynthetic proteins and complexes.

### *PGA1* encodes AtFtsH12

**Figure 2:**
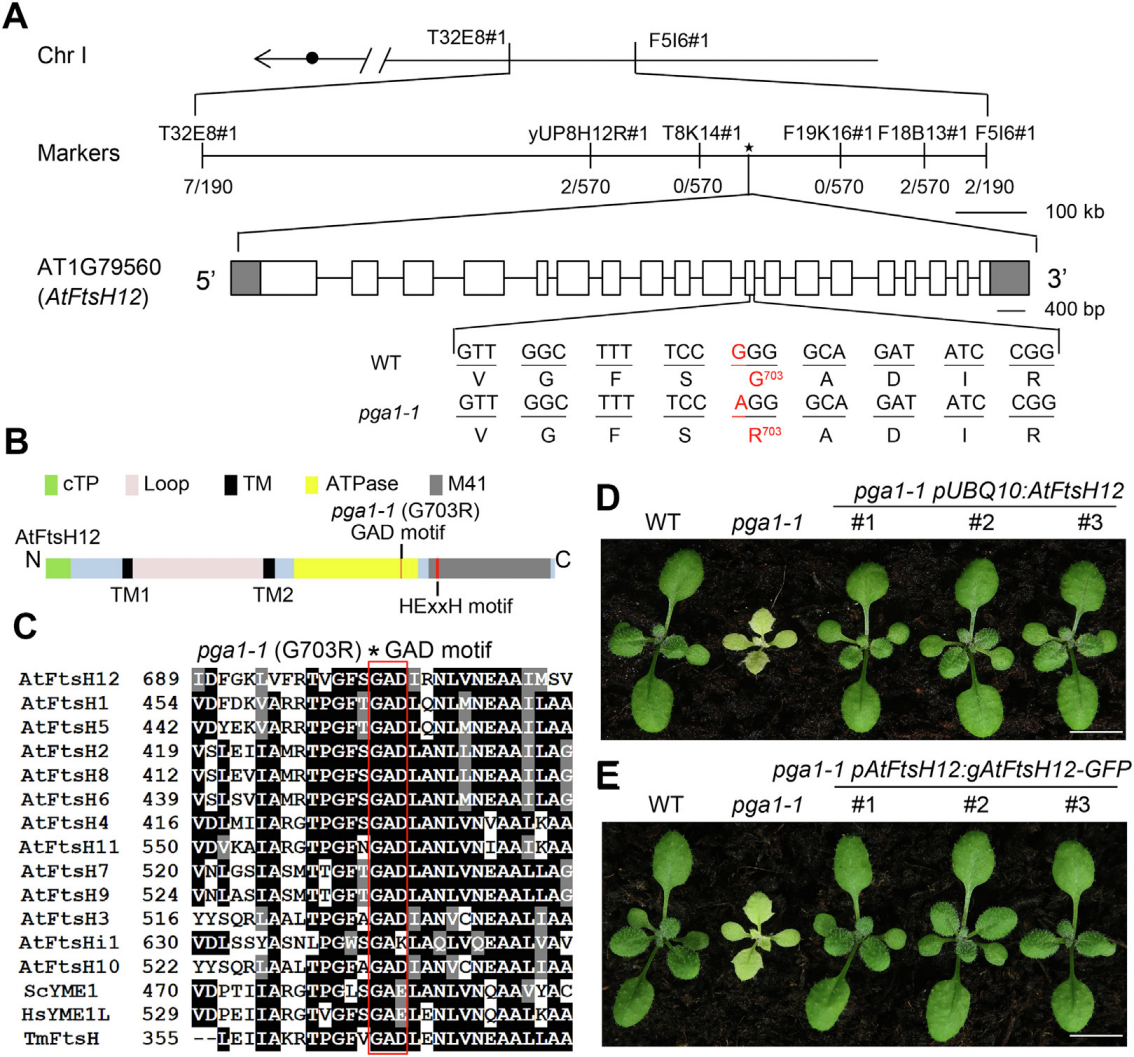
***PGA1* encodes AtFtsH12.***A*, map-based cloning of the *PGA1* locus. The *PGA1* was mapped between molecular markers *T8K14#1* and *F19K16#1* using 285 individual plants. In this region, a G703R mutation in AT1G79560/AtFtsH12 was identified. *B*, schematic representation of protein domains in AtFtsH12. *C*, the GAD motif is highly conserved in FtsH homologs, including 12 AtFtsHs from *Arabidopsis thaliana*, ScYME1 from *Saccharomyces cerevisiae*, HsYME1L from *Homo sapiens*, and TmFtsH from *Thermotoga maritime*. *D*, complementation of *pga1-1* with *pUBQ10:AtFtsH12*. *E*, complementation of *pga1-1* with *pAtFtsH12:gAtFtsH12-GFP*. Representative 2-week-old plants are shown in (*D*) and (*E*). The scale bars represent 0.5 cm. ATPase, the ATPase domain; cTP, chloroplast transit peptide; GAD, the Gly-Ala-Asp motif in the ATPase domain; Loop, the loop domain between TM1 and TM2; M41, Zinc-dependent protease M41 domain; TM, putative transmembrane domains.

To identify the *PGA1* locus, a map-based cloning approach was employed, using a F2 population derived from a cross between *pga1-1* and the Landsberg *erecta* (L*er*) ecotype. Initial mapping was performed with a DNA pool from 95 *pga1-1* individuals using molecular markers distributed on five chromosomes, and the *PGA1* locus was located to the long arm of chromosome 1, close to the marker *F5A18#1* (Fig. S3). Fine mapping using 285 *pga1-1* individuals further placed *PGA1* between markers *T8K14#1* and *F19K16#1* (Fig. 2*A*). Genomic DNA sequencing of genes in this region identified a G to A missense mutation in the coding region of AT1G79560, converting the 703rd amino acid residue from glycine to arginine (G703R) (Fig. 2*A*). AT1G79560 encodes AtFtsH12, a member of the chloroplast FtsH protein family (22). AtFtsH12 protein contains several distinct domains including an N-terminal chloroplast transit peptide, two putative transmembrane domains TM1 and TM2, an ATPase domain, and a conserved HExxH motif for the zinc-dependent proteolytic domain M41 (Figs. 2*B*, S4 and S5). The G703R mutation was located in the GAD (Glycine-Alanine-Aspartate/Glutamate) motif, which is part of the ATPase domain and is well conserved in FtsH homologs (Figs. 2, *B* and *C* and S4) (37). The predicted 3-D structure of the ATPase domain of AtFtsH12 showed that the conserved Gly703 is in an α-helix near the ATP-binding pocket, resembling its counterpart Gly484 in yeast Yme1 (Figs. 2*C* and S4) (34).

To confirm that *PGA1* is *AtFtsH12*, we complemented the *pga1-1* mutant with two constructs. One vector contained the full-length *AtFtsH12* cDNA driven by the constitutive *UBQ10* promoter (*pUBQ10:AtFtsH12*), while the other included the full-length *AtFtsH12* genomic region fused with GFP-coding sequences at the 3′ driven by its native promoter (*pAtFtsH12:gAtFtsH12-GFP*) (Fig. 2, *D* and *E*). Independent *pga1-1 pUBQ10:AtFtsH12* and *pga1-1 pAtFtsH12:gAtFtsH12-GFP* transgenic lines showed WT-like green leaf color and plant stature, indicating that *pga1-1* can be rescued by *AtFtsH12* and that AtFtsH12-GFP is functional *in planta* (Fig. 2, *D* and *E*). To detect the AtFtsH12 protein, we raised a polyclonal antibody against the loop domain between the two transmembrane domains TM1 and TM2 of AtFtsH12 (Fig. 2*B*). In *pga1-1 pAtFtsH12:gAtFtsH12-GFP* lines, the AtFtsH12-GFP fusion protein (∼130 kDa) was readily detected using either an anti-GFP antibody (Fig. S6*A*) or the anti-AtFtsH12 antibody (Fig. S6, *B* and *C*). The endogenous AtFtsH12 (∼100 kDa) was also detected by the anti-AtFtsH12 antibody (Fig. S6, *B* and *C*). Together, these results demonstrate that the mutation in *AtFtsH12* causes the *pga1-1* phenotype, and *PGA1* encodes AtFtsH12.

Null mutants of *AtFtsH12* were previously reported as embryonic lethal (30, 31, 33). Thus, the viable *pga1-1* represents a novel hypomorphic allele of *AtFtsH12* and provides a valuable material to dissect *AtFtsH12* functions. In addition, when *pUBQ10:AtFtsH12* or *pAtFtsH12:gAtFtsH12-GFP* was transformed into WT or *pga1-1*, ∼20% of hygromycin-resistant T1 transgenic plants showed different degrees of albino leaf coloration and abnormal leaf development in rosette leaves (Fig. S7*A*). We examined the transcript and the protein levels of AtFtsH12 in T1 albino transgenic lines. Surprisingly, *AtFtsH12* transcripts were highly accumulated in the white tissues, but AtFtsH12 protein level was greatly reduced compared with those in WT (Fig. S7, *B* and *C*). This suggests that the albino transgenic plants may not arise from reduced *AtFtsH12* mRNA levels due to cosuppression. Alternative possibilities, such as poisonous effects of overexpressed *AtFtsH12*, may be responsible for this phenotype. Together with the pale-green phenotype of *pga1-1*, these results indicate that AtFtsH12 plays essential roles in chloroplast development during vegetative growth.

### AtFtsH12 forms large protein complexes on the chloroplast envelope

**Figure 3:**
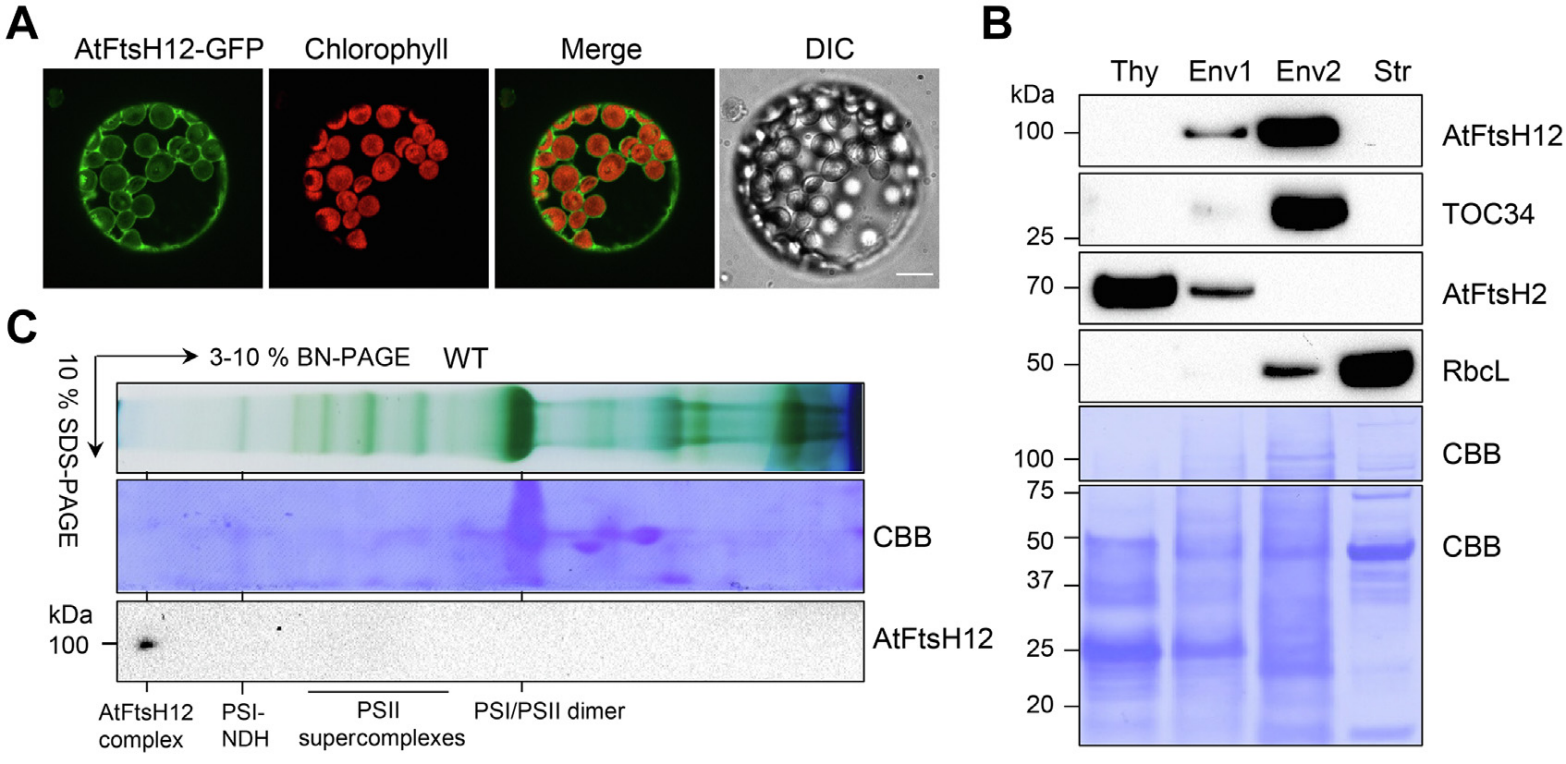
**AtFtsH12 forms large protein complexes on the chloroplast envelope.***A*, protoplasts were prepared from *pga1-1 pAtFtsH12:gAtFtsH12-GFP*. GFP and chlorophyll fluorescence were detected by confocal microscopy. The differential interference contrast (DIC) image confirmed the intactness of protoplasts. The scale bar represents 10 μm. *B*, intact chloroplasts were fractionated into thylakoid, Env1 (mixed envelopes and thylakoids), Env2 (mainly envelopes), and the stroma. TOC34, AtFtsH2, and RbcL served as markers for the envelope, thylakoids, and the stroma respectively. Protein loading was normalized to equal protein amount and confirmed by CBB-stained PVDF membranes. *C*, the 1-D BN-PAGE and the 2-D SDS-PAGE were performed as in Figure 1*E*. Migrations of PSII supercomplexes and PSI-NDH supercomplexes were indicated (38). BN-PAGE, blue native PAGE; CBB, Coomassie Brilliant Blue; PSI, Photosystem I; PSII, Photosystem II.

AtFtsH12 is predicted to contain a chloroplast transit peptide, and we used two methods to determine the subcellular localization of AtFtsH12. First, leaf mesophyll protoplasts were isolated from the *pga1-1 pAtFtsH12:gAtFtsH12-GFP* lines, and ring-like AtFtsH12-GFP signals nicely surrounding the chlorophyll fluorescence were observed, indicating that AtFtsH12-GFP is targeted to the chloroplast and is likely localized to the chloroplast envelope membrane (Figs. 3*A* and S6*D*). Interestingly, some GFP signals were also observed in the cytosol (Figs. 3*A* and S6*D*). Immunoblotting using the anti-GFP antibody revealed a protein band (∼37 kDa) in *pga1-1 pAtFtsH12:gAtFtsH12-GFP* (Fig. S6*A*). Therefore, the cytosol GFP signals may derive from degraded AtFtsH12-GFP fragments that were not recognized by the anti-AtFtsH12 antibody against the loop structure of AtFtsH12 (Figs. 2*B* and S6*C*).

As an alternative approach, we fractionated intact chloroplasts from WT plants into Thy (thylakoids), Env1 (mixed thylakoid and envelope membranes), Env2 (mostly envelope membranes), and Str (stroma) as described (35). Equal amounts of proteins from these fractions were analyzed with immunoblotting against different marker proteins, including VAR2/AtFtsH2 for thylakoids, TOC34 (Translocase of chloroplast 34) for envelope, and RbcL for stroma respectively, confirming the identities of these fractions (Fig. 3*B*). AtFtsH12 was mainly detected in Env2, as well as a minor presence in Env1 using the anti-AtFtsH12 antibody, supporting that AtFtsH12 is associated with the chloroplast envelope (Fig. 3*B*). Next, we checked whether AtFtsH12 forms protein complexes like other Arabidopsis FtsH proteins (22, 24, 26). Immunoblotting of the 2-D SDS-PAGE gel showed that AtFtsH12 was present in high molecular weight complexes that were significantly larger than known photosynthetic protein complexes, such as PSII supercomplexes and PSI-NDH supercomplexes (Fig. 3*C*) (38). These large AtFtsH12 complexes are consistent with previously reported 2-MDa AtFtsH12 complexes that are associated with the TIC complexes (30).

### The feedback regulation of *AtFtsH12* gene expression by the *pga1-1* mutation

**Figure 4:**
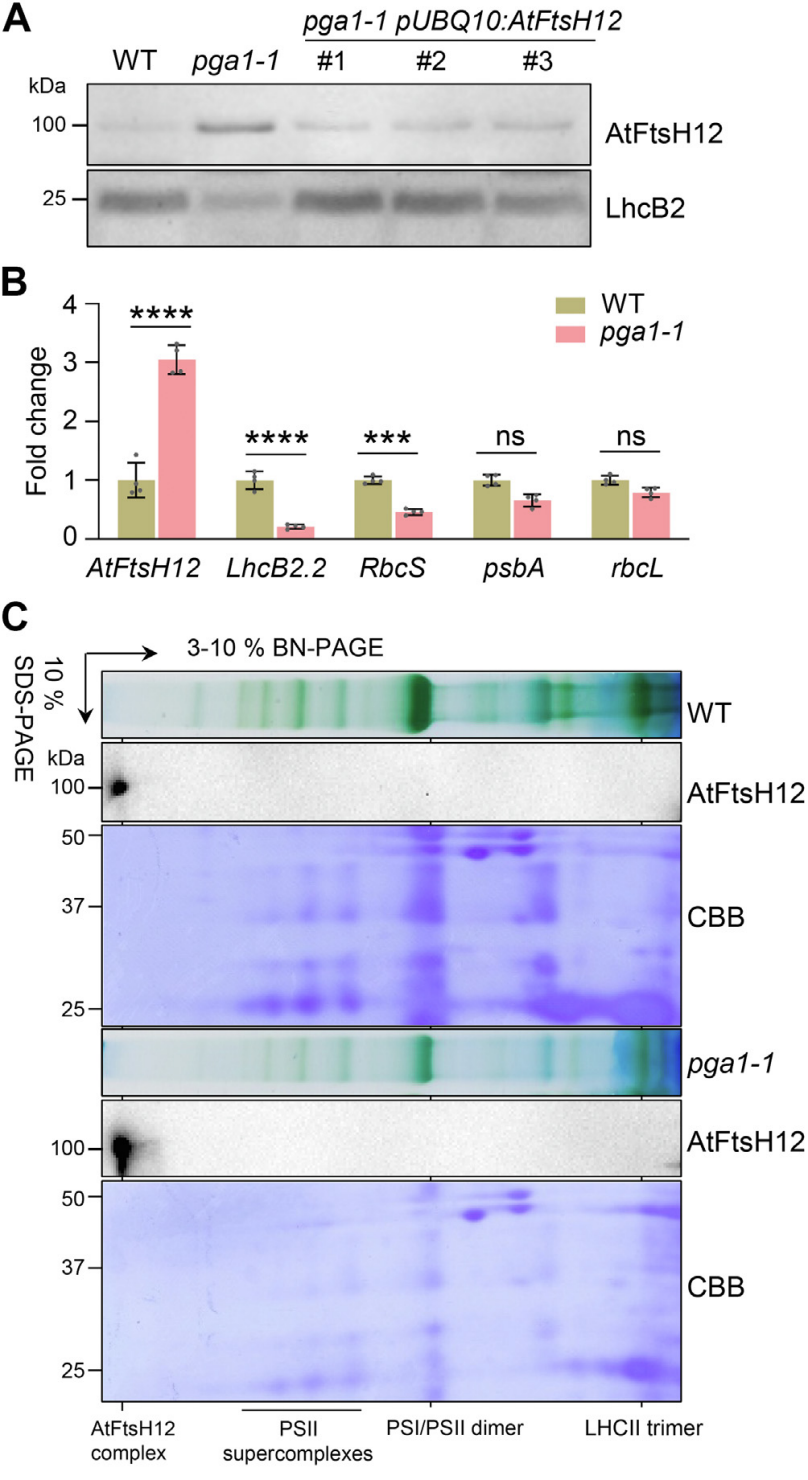
**The feedback regulation of *AtFtsH12* expression in *pga1-1*.***A*, the accumulation of AtFtsH12 and LhcB2 in 2-week-old WT, *pga1-1*, and *pga1-1 pUBQ10:AtFtsH12*. Total proteins were extracted, and protein loading was normalized to equal fresh tissue weight. *B*, RT-qPCR analyses of the steady-state transcript levels of *AtFtsH12*, *LhcB2.2*, *RbcS*, *psbA*, and *rbcL*. Relative transcript levels with respect to those in the WT were calculated using the 2^−ΔΔCt^ method, and *Actin2* was used as the reference gene. Data are means ± s.d. of four biological replicates. ns not significant, ∗∗∗0.0001 < *p* < 0.001, and ∗∗∗∗*p* < 0.0001. *C*, AtFtsH12^G703R^ mutant proteins are assembled into large protein complexes in *pga1-1*. The 1-D BN-PAGE and 2-D SDS-PAGE were performed as in Figure 1*E*. BN-PAGE, blue native PAGE.

In immunoblotting analyses, we consistently observed that AtFtsH12^G703R^, the mutant form of AtFtsH12, accumulated to a higher level in *pga1-1* than the level of AtFtsH12 in the WT (Figs. 4*A* and 6, *B* and *C*). The increased accumulation of AtFtsH12^G703R^ was restored to the WT level in *pga1-1 pUBQ10:AtFtsH12* and *pga1-1 pAtFtsH12:gAtFtsH12-GFP*, respectively (Figs. 4*A* and S6, *B* and *C*). Interestingly, the transcript level of *AtFtsH12*^*G703R*^ was also elevated in *pga1-1* revealed by RT-qPCR (Fig. 4*B*). In contrast, the transcript levels of *PhANGs* such as *LhcB2.2* and *RbcS* were significantly reduced (Fig. 4*B*). However, the transcript levels of chloroplast genome-encoded photosynthetic genes such as *psbA* and *rbcL* in *pga1-1* were comparable to those in the WT (Fig. 4*B*). Using BN-PAGE, we observed that the G703R mutation did not affect the formation of AtFtsH12 complexes, and AtFtsH12^G703R^ complexes also accumulated to a markedly higher level in *pga1-1* than the level of AtFtsH12 complexes in the WT, indicating that AtFtsH12^G703R^ could be assembled into large complexes effectively (Fig. 4*C*).

To establish the genetic basis for this feedback regulation, heterozygous *pga1-1* mutants (*pga1-1*/+) were examined. *pga1-1*/+ showed WT-like phenotypes, and the accumulation of AtFtsH12 protein in *pga1-1*/+ was similar to that of the WT (Fig. S8*C*). These findings suggest that *pga1-1* is a recessive mutant, and the feedback regulation of *AtFtsH12* is not activated in *pga1-1* heterozygotes.

### AtFtsH12 is required for chloroplast development and the accumulation of cytosol-translated chloroplast proteins

**Figure 5:**
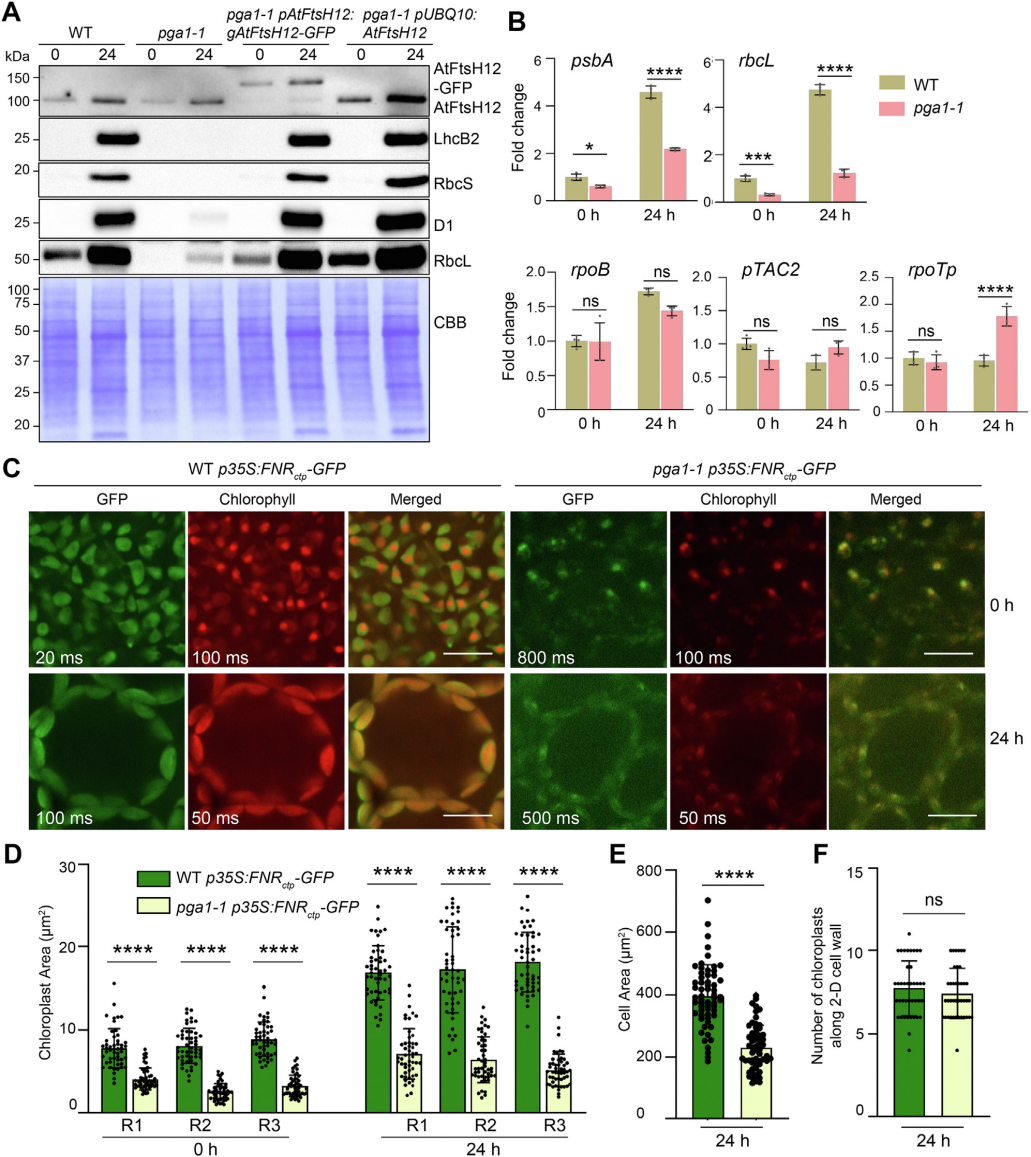
**AtFtsH12 is required for chloroplast protein homeostasis and chloroplast development.***A*, the accumulation of photosynthetic proteins and AtFtsH12 in WT, *pga1-1*, *pga1-1 pAtFtsH12:gAtFtsH12-GFP*, and *pga1-1 pUBQ10:AtFtsH12* before and after 24-h de-etiolation. Protein loading was normalized to equal fresh tissue weight and confirmed by CBB-stained PVDF membranes. *B*, RT-qPCR analyses of the steady-state transcript levels of *psbA*, *rbcL*, *rpoB*, *pTAC2*, and *rpoTp* in WT and *pga1-1* before and after 24-h de-etiolation. Relative transcript levels with respect to those in the WT were calculated using the 2^−ΔΔCt^ method, and *PP2A* was used as the reference gene. Data are means ± s.d. of four biological replicates. ns not significant, ∗0.01 < *p* < 0.05, ∗∗∗0.0001 < *p* < 0.001, and ∗∗∗∗*p* < 0.0001. *C*, the accumulation of GFP in chloroplasts of WT *p35S:FNR*_*ctp*_*-GFP* and *pga1-1 p35S:FNR*_*ctp*_*-GFP* before and after 24-h de-etiolation. The scale bars represent 10 μm. GFP and chlorophylls (protochlorophyllide at 0 h) were detected by confocal microscopy. Exposure times in milliseconds (ms) were labeled in order to compare fluorescent signal intensities in different genotypes. *D*, changes of chloroplast area during de-etiolation for 24 h. Three replicates containing 150 chloroplasts were analyzed (n = 50 chloroplasts in each replicate) for each genotype. *E*, cell area after 24-h de-etiolation. Three replicates containing 60 cells were analyzed (n = 20 cells in each replicate) for each genotype. *F*, average number of chloroplasts along the 2-D cell wall after 24-h de-etiolation (n = 40 cells were counted in each genotype). ns not significant, and ∗∗∗∗*p* < 0.0001. CBB, Coomassie Brilliant Blue.

To further analyze the accumulation of photosynthetic proteins in *pga1-1*, we used a de-etiolation assay, which enables the determination of photosynthetic protein levels in a time course during illumination (29). The protein level of AtFtsH12 increased in the WT upon 24-h illumination, indicating that AtFtsH12 is induced during de-etiolation (Figs. 5*A* and S9). Interestingly, the accumulation of the mutant form of AtFtsH12 was not elevated in etiolated *pga1-1* seedlings compared with that of the WT form of AtFtsH12 in etiolated WT seedlings, in contrast to the findings with 2-week old light-grown WT and *pga1-1* seedlings (Figs. 5*A* and 4*A*). Upon 24-h light treatment, the level of the mutant form of AtFtsH12 was still comparable in *pga1-1* compared with that of the WT form of AtFtsH12 in the WT (Fig. 5*A*). In addition, the accumulation of chloroplast genome-encoded photosynthetic proteins, such as RbcL and D1, as well as nuclear genome-encoded photosynthetic proteins RbcS and LhcB2, were also strongly induced during de-etiolation in the WT (Fig. 5*A*). In stark contrast, the accumulation of these proteins was greatly compromised in *pga1-1* during de-etiolation, and the defective accumulation of photosynthetic proteins in *pga1-1* was recovered in complementation lines (Fig. 5*A*). At the mRNA level, PEP-dependent transcripts such as *rbcL* and *psbA* accumulated to much lower levels in *pga1-1* compared with those in the WT after 24-h illumination, suggesting that abnormal accumulation of photosynthetic proteins was associated with defective chloroplast gene expression (Fig. 5*B*). However, the NEP-transcribed gene *rpoB* was only modestly affected in *pga1-1* during de-etiolation (Fig. 5*B*). The expression of *pTAC2*, a nuclear gene encoding an accessory subunit of PEP involved in the assembly of PEP complexes (39), was not affected in *pga1-1* (Fig. 5*B*). Interestingly, the *rpoTp* gene, encoding the NEP, was upregulated in *pga1-1* (Fig. 5*B*).

To observe cytosol-translated chloroplast protein accumulation *in vivo* during de-etiolation, we introduced a *p35S:FNR*_*ctp*_*-GFP* chloroplast-localized GFP reporter line into *pga1-1* (40) to generate *pga1-1 p35S:FNR*_*ctp*_*-GFP*. As the expression of *FNR*_*ctp*_*-GFP* was driven by the constitutive *35S* promoter, signals of FNR_ctp_-GFP were observed in etioplasts (at 0 h) and chloroplasts (at 24-h illumination) in the WT background (Fig. 5*C*). In contrast, the accumulation of FNR_ctp_-GFP signals was severely reduced in etioplasts and chloroplasts in *pga1-1* (Fig. 5*C*). In addition, we observed that protochlorophyllide (0 h) and chlorophyll (24 h) fluorescence in *pga1-1* were also severely reduced compared with those in the WT (Fig. 5*C*). Moreover, the sizes of etioplasts (0 h) and chloroplasts (24 h) were smaller in *pga1-1* than those in the WT (Fig. 5*D*). Average mesophyll cell area in cotyledons was also significantly reduced in *pga1-1* compared with that in the WT (Fig. 5*E*). However, the average number of chloroplasts per cell was comparable in *pga1-1* and WT (Fig. 5*F*). These data suggest that the partial loss of AtFtsH12 function leads to defective chloroplast development and reduced accumulation of chloroplast proteins during de-etiolation.

**Figure 6:**
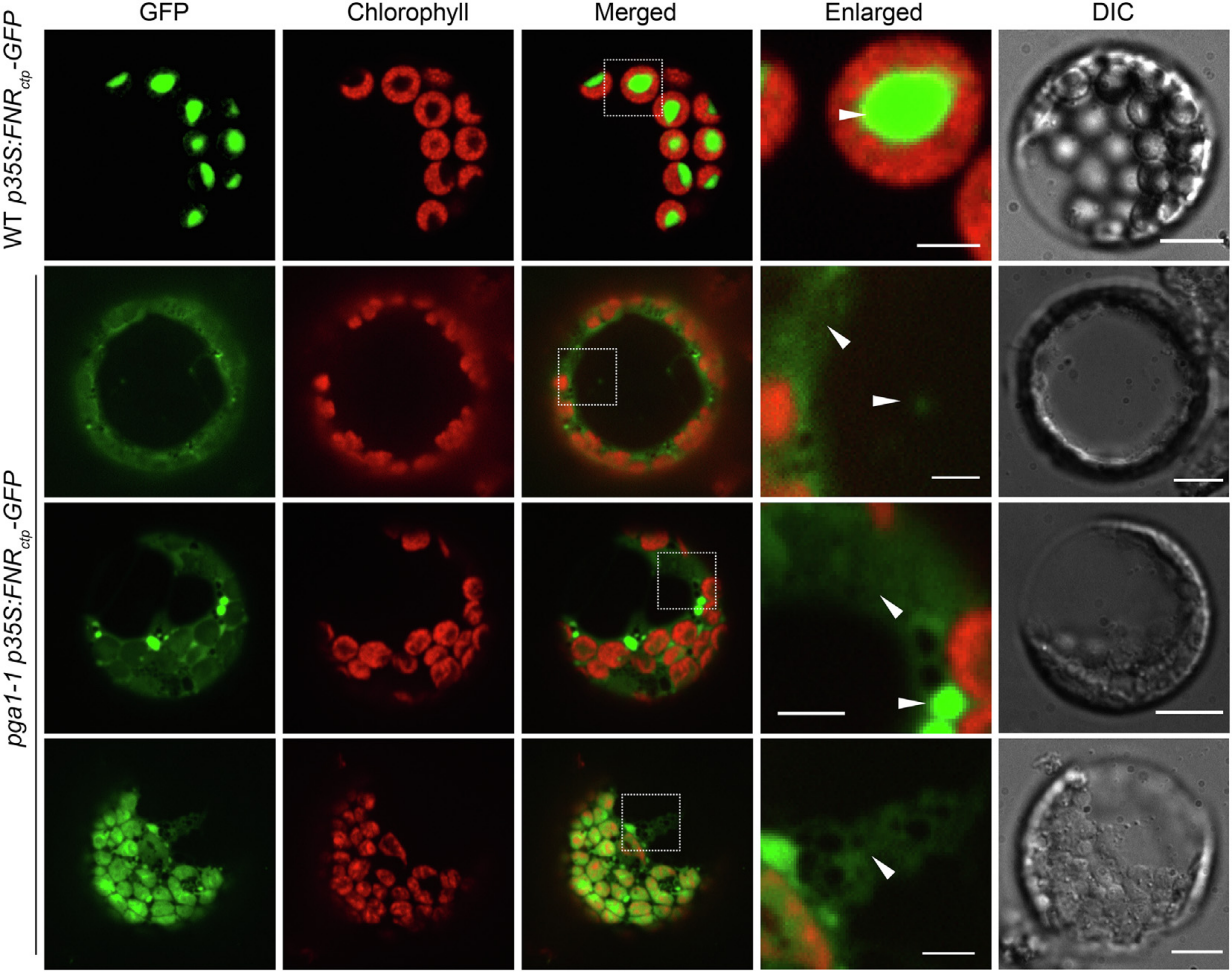
**The accumulation of FNR**_**ctp**_**-GFP in *pga1-1*.** Protoplasts were isolated from rosette leaves of 4-week-old WT *p35S:FNR*_*ctp*_*-GFP* and *pga1-1 p35S:FNR*_*ctp*_*-GFP*. GFP and chlorophyll fluorescent signals were detected by confocal microscopy. *White boxes* indicate the enlarged areas. *White arrows* show the GFP signals that did not overlap with chloroplasts. The scale bars represent 10 μm in DIC images. The scale bars represent 2.5 μm in enlarged images.

Next, we investigated the accumulation of FNR_ctp_-GFP in protoplasts isolated from rosette leaves of WT *p35S:FNR*_*ctp*_*-GFP* and *pga1-1 p35S:FNR*_*ctp*_*-GFP*, respectively (Fig. 6). In WT *p35S:FNR*_*ctp*_*-GFP*, strong FNR_ctp_-GFP signals were exclusively observed in the stroma of chloroplasts (Fig. 6). In contrast, FNR_ctp_-GFP showed much reduced signals in the chloroplast in *pga1-1 p35S:FNR*_*ctp*_*-GFP* protoplasts, while an aberrant accumulation of FNR_ctp_-GFP signals in the cytosol was also observed in *pga1-1 p35S:FNR*_*ctp*_*-GFP* protoplasts (Fig. 6). These findings suggest that AtFtsH12 is required for the accumulation of cytosol-translated chloroplast proteins in the chloroplast.

To investigate how AtFtsH12 modulates the accumulation of PEP complexes, we introduced *pTAC2-GFP* fusion gene driven by the native *pTAC2* promoter (*PropTAC2:pTAC2-GFP*) into the *pga1-1* background by genetic crossing. In 4-day-old cotyledons, signals of chlorophyll autofluorescence were much weaker in *pga1-1 PropTAC2:pTAC2-GFP* lines than in the *ptac2-5 PropTAC2:pTAC2-GFP* complementation lines (Fig. S10), suggesting that chloroplasts in *pga1-1* were underdeveloped. Signals of pTAC2-GFP were also much lower in the *pga1-1 PropTAC2:pTAC2-GFP* lines than in the *ptac2-5 PropTAC2:pTAC2-GFP* lines (Fig. S10). These data demonstrate that AtFtsH12 is involved in the accumulation of the pTAC2 subunit of the PEP complex.

### The *pga1-1* mutation triggers cytosolic and chloroplast protein stress responses

In contrast to the canonical bacterial FtsH harboring a linker region of ∼70 amino acid residues between the two transmembrane domains, AtFtsH12 contains a longer linker region with ∼260 amino acid residues, which are highly conserved in AtFtsH12 homologs from *Chara braunii*, *Klebsormidium nitens*, *Marchantia polymorpha*, and *Physcomitrella patens* (Figs. 2*B* and S11*A*). Based on the topology of AtFtsH12, this loop is predicted to be located in the intermembrane space between the outer and inner envelopes (30, 31, 32). Yeast two-hybrid assay showed that the loop domain of AtFtsH12 can interact directly with the N-terminus of LhcB2 precursor protein (Fig. S11, *B* and *C*). When the chloroplast import apparatus was disrupted and LhcB2 precursors were overaccumulated in the cytosol, the expression of *ctHSP70*, coding for a cytosol-localized HSP70, was induced in response to cytosolic protein stress (11).

**Figure 7:**
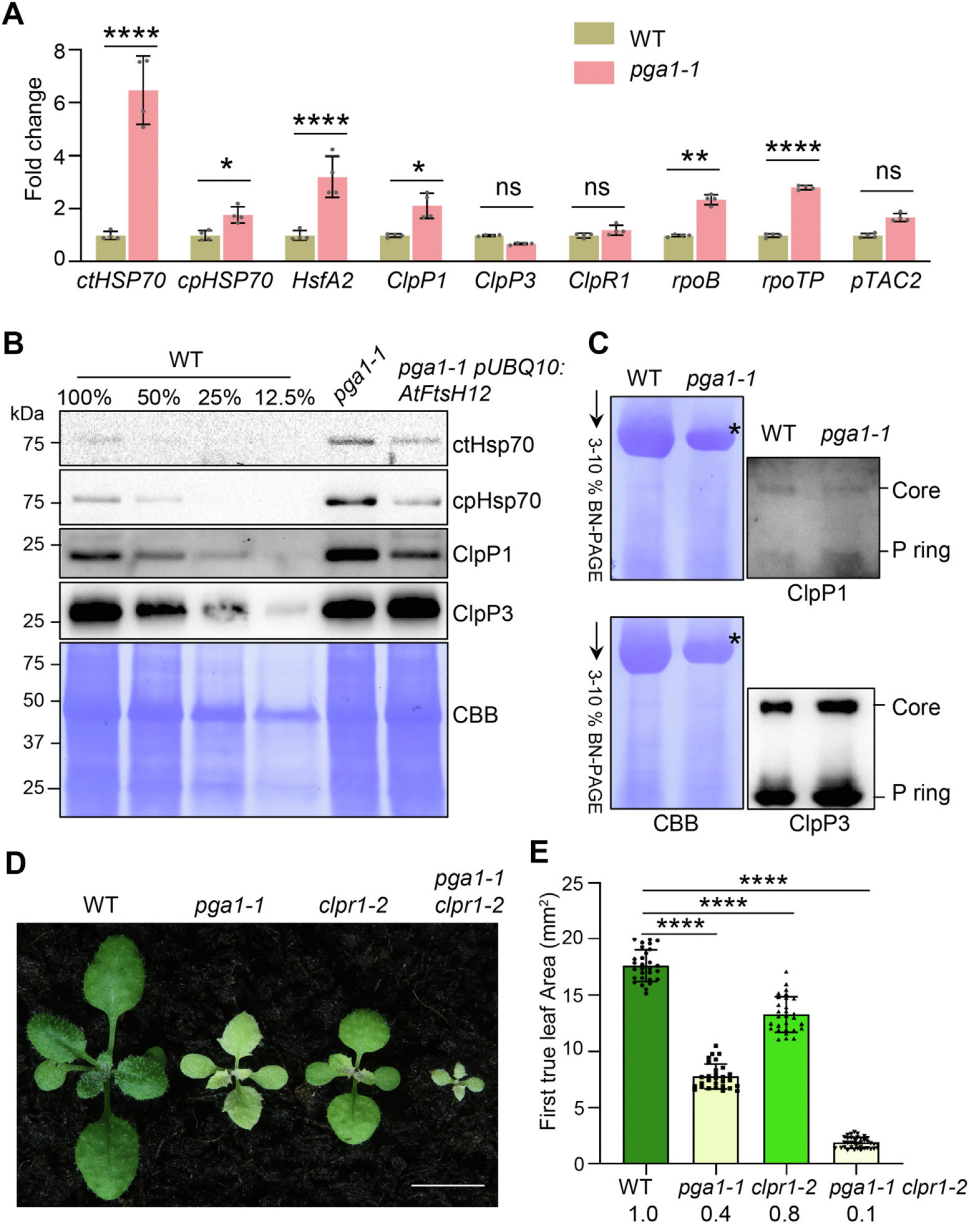
**The *pga1-1* mutation triggers cytosolic and chloroplast protein stress responses.***A*, RT-qPCR analyses of steady-state transcript levels of *ctHSP70*, *cpHSP70*, *HsfA2*, *ClpP1*, *ClpP3*, *ClpR1*, *rpoB*, *rpoTP*, and *pTAC2* in 2-week-old WT and *pga1-1*. Relative transcript levels with respect to those in the WT were calculated using the 2^−ΔΔCt^ method, and *Actin2* was used as the reference gene. Data are means ± s.d. of four biological replicates. ns not significant, ∗0.01 < *p* < 0.05, ∗∗0.001 < *p* < 0.01, and ∗∗∗∗*p* < 0.0001. *B*, the accumulation of ctHSP70, cpHSP70, ClpP1, and ClpP3 in 2-week-old WT, *pga1-1*, and *pga1-1 pUBQ10:AtFtsH12*. Total proteins were extracted, and protein loading was normalized to equal fresh tissue weight and confirmed by CBB-stained PVDF membranes. *C*, the accumulation of Clp complexes in 2-week-old WT and *pga1-1*. Soluble proteins were extracted and resolved on 1-D BN-PAGE (*left panel*). The 1-D gel immunoblotting was performed using anti-ClpP1 and anti-ClpP3 antibodies (*right panel*). The Core complex and P ring complexes were indicated according to the migration of RuBisCO (indicated by the *asterisk*) (65). *D*, phenotypes of 2-week-old WT, *pga1-1*, *clpr1-2*, and *pga1-1 clpr1-2*. The scale bar represents 0.5 cm. *E*, relative leaf areas of the first pair of rosette leaves of 2-week-old WT, *pga1-1*, *clpr1-2*, and *pga1-1 clpr1-2* (n ≥ 29 individuals for each genotype). The average leaf area of the WT was defined as 1.0. ∗∗∗∗*p* < 0.0001. BN-PAGE, blue native PAGE; CBB, Coomassie Brilliant Blue; RuBisCO, ribulose-1,5-bisphosphate carboxylase/oxygenase.

We reasoned that the compromised accumulation of *PhANG* products in chloroplast may induce a similar response in *pga1-1*. At the transcript level, we found that both *ctHSP70* and *cpHSP70* coding for a chloroplast-localized HSP70 were upregulated in *pga1-1* compared with the WT (Fig. 7*A*). In addition, the expression of *HsfA2*, encoding the Heat Shock Transcription Factor A2 (HsfA2) involved in the chloroplast-unfolded protein response (cpUPR) (15, 16), was also upregulated in *pga1-1* (Fig. 7*A*). Consistent with transcript levels, ctHSP70 and cpHSP70 proteins accumulated to higher levels in *pga1-1* (Fig. 7*B*). The ectopic induction of ctHSP70 and cpHSP70 was reversed in *pga1-1 pUBQ10:AtFtsH12* lines (Fig. 7*B*). Chloroplast proteases are also involved in maintaining proteostasis during cpUPR (10). The Clp complex is a major protease system involved in the degradation of chloroplast proteins (18). We found that the accumulation of ClpP1 was increased in *pga1-1* compared with that in the WT, but ClpP3 level was not significantly altered (Fig. 7*B*). The increase of ClpP1 protein level is consistent with the upregulation of its transcripts (Fig. 7*A*). Next, we analyzed the accumulation of Clp complexes in WT and *pga1-1* (Fig. 7*C*). In contrast to a greatly reduced level of RuBisCO in *pga1-1*, the levels of Clp complexes including the core complex and the P ring complex were not affected (Fig. 7*C*). These findings suggest that Clp complexes are maintained in *pga1-1*, despite defective chloroplast development.

To probe the genetic interaction between *AtFtsH12* and components in chloroplast protein stress response, we generated double mutants of *pga1-1* and *clpr1-2* (SALK_088407), a T-DNA insertion allele of *ClpR1*, encoding a nonproteolytic subunit of the Clp complex (41, 42). *clpr1-2* displayed a distinct virescent leaf color phenotype and a smaller plant stature (Fig. 7*D*). In *pga1-1 clpr1-2* double mutant, we observed a dramatic reduction of rosette leaf sizes compared with the respective single mutants (Fig. 7*D*). Quantitative analysis revealed that the average sizes of the first pair of rosette leaves in *pga1-1* and *clpr1-2* was ∼40% and ∼80% of that in the WT, respectively (Fig. 7*E*). In contrast, the average size of the first pair of rosette leaves in *pga1-1 clpr1-2* was only ∼10% of that in the WT, significantly smaller than the expected leaf size if the genetic interaction between *pga1-1* and *clpr1-2* is additive (0.8 × 0.4 = 0.32) (Fig. 7*E*) (43). These findings suggest a synergistic genetic interaction between *PGA1*/*AtFtsH12* and *ClpR1* in regulating plant development.

Together, our data show that a hypomorphic mutation in *PGA1*/*AtFtsH12* leads to the induction of cytosol and chloroplast protein stress responses, and chloroplast protein accumulation and degradation coordinately regulate chloroplast and plant development.

## Discussion

FtsH proteins are conserved membrane-localized, ATP-dependent AAA+ family members that play essential roles in protein quality control in both prokaryotes and eukaryotes (21, 44). The *Arabidopsis thaliana* genome encodes 12 AtFtsH proteins that are localized to the membrane systems of chloroplasts and mitochondria (22). Despite the tremendous progress in the elucidation of AtFtsH functions, much remains to be learned about the regulation of organelle functions by AtFtsH proteins. We have been investigating the regulation of chloroplast development by the thylakoid FtsH complexes, using the *var2* leaf variegation mutant which is defective in VAR2/AtFtsH2 (45, 46). Extensive characterizations of *var2* genetic modifier mutants established that the cytosol-chloroplast proteostasis can regulate leaf variegation and chloroplast development (29, 47). In this work, we reported the isolation of the *pale green Arabidopsis 1-1* (*pga1-1*) mutant. We confirmed that *pga1-1* was caused by a point mutation in *PGA1*/*AtFtsH12*, encoding a member of the AtFtsH family proteins (22). *AtFtsH12* is an essential gene in Arabidopsis and previous work has shown that null alleles of *AtFtsH12* are embryonic lethal (30, 31, 32), thus the hypomorphic *pga1-1* allele offers a unique and valuable genetic resource for the investigation of AtFtsH12 functions.

Interestingly, *pga1-1* contains a G703R mutation in the conserved GAD motif of the ATPase domain (Fig. 2*C*). A similar glycine to arginine mutation (G433R) in the GAD motif of VAR2/AtFtsH2 was also reported in the *var2-19* mutant, which causes a leaf variegation phenotype in *Arabidopsis* (37). These findings on one hand show that the conserved glycine is important for AtFtsH12 and VAR2/AtFtsH2 functions but on the other hand indicate that the Gly to Arg mutations likely do not abolish protein functions entirely. This raises a tempting implication that a similar Gly to Arg genetic manipulation may provide a strategy to bypass the lethal phenotype of other essential proteins containing the conserved ATPase domain. Unexpectedly, the G703R mutation in AtFtsH12 does not affect the assembly and the size of AtFtsH12 complexes in *pga1-1* (Fig. 4*C*), which exists in large protein complexes in agreement with previous reports (30). Together with published results, our findings suggest that the G703R mutation leads to a reduced but not completely abolished AtFtsH12 activity (34, 37).

In addition to the AAA+ ATPase domain, many FtsH proteins also contain a conserved HExxH M41 metalloprotease domain (Fig. S5). The proteolytic activities of FtsH family members have been demonstrated in a number of cases (48, 49, 50). However, despite the presence of the conserved HExxH sequences in AtFtsH12, the conserved histidine^769^ in the proteolytic motif M41 is dispensable for chloroplast and plant development (30). Similarly, the conserved histidine^488^ is also dispensable for VAR2/AtFtsH2 in chloroplast development (51). It had been proposed that ATP-dependent FtsH proteins not only mediate proteolysis but also the insertion of proteins into membranes and assembly of protein complexes, acting like a chaperone (52, 53, 54). The participations of FtsHs in protein transport and assembly into membranes have been reported in *Escherichia coli* and *Arabidopsis* (30, 55). Similar to its homologs function as a chaperone in bacteria, it was proposed that AtFtsH12, together with other AtFtsHi members, may provide driving forces to pull chloroplast protein precursors through envelope membranes by the hydrolysis of ATP with the ATPase domain (30). It is also speculated that AtFtsH12 may have similar functions in organellar membrane protein quality control as its homologs. Whether AtFtsH12 acts on misfolded or unassembled proteins in the chloroplast envelope requires further investigation.

Several lines of evidence support the notion that AtFtsH12 is required for the accumulation of chloroplast proteins. Firstly, we determined that the steady levels of many nuclear-encoded and plastid-encoded photosynthetic proteins were reduced significantly in *pga1-1* compared with the WT (Fig. 1*D*). Moreover, the accumulation of most photosynthetic protein complexes was also reduced in *pga1-1* (Fig. 1, *E* and *F*). Overall, the most dramatic impact of the loss of AtFtsH12 seems to be on the accumulation of LHCs and their subunits. Consistently, we observed that the loop domain between the transmembrane domains TM1 and TM2 of AtFtsH12 was able to interact with LhcB2 in yeast two-hybrid assays, suggesting a direct functional link between the two proteins (Fig. S11). Alternatively, as the most abundant membrane proteins in the chloroplast, LHC protein accumulation may be more sensitive to disturbed chloroplast protein homeostasis (56). Secondly, the accumulation of photosynthetic proteins was examined in de-etiolation assays. The *pga1-1* mutant showed a greatly compromised photosynthetic protein accumulation during de-etiolation (Fig. 5*A*). Thirdly, we monitored the accumulation of two chloroplast GFP marker proteins in the chloroplast in the *pga1-1* mutant. *FNR*_*ctp*_*-GFP* driven by the *35S* constitutive promoter and *pTAC2-GFP* driven by its native promoter were not efficiently accumulated in the chloroplast in *pga1-1* (Figs. S10 and 5*C*). In addition, we observed some GFP signals that did not overlap with chlorophylls in protoplasts of *pga1-1 p35S:FNR*_*ctp*_*-GFP* (Fig. 6). The compromised accumulation of chloroplast proteins and GFP reporters in *pga1-1* might be caused by various mechanisms, including gene transcription, protein translation and translocation, stability, assembly and so on.

The aberrant induction of *AtFtsH12* transcripts in *pga1-1* indicates the possible operation of feedback regulation of *AtFtsH12* by its translation product and the WT form of AtFtsH12 may be part of a pathway that represses its own expression (Fig. 4*A*). Alternatively, it is also plausible that the induced *AtFtsH12* expression represents a compensating response to the defective chloroplast protein accumulation and chloroplast development in *pga1-1*. However, this feedback regulation of the accumulation of AtFtsH12 in *pga1-1* was not observed during the 24-h de-etiolation assay, suggesting that this mechanism may be regulated by developmental stages or growth conditions (Figs. 4*A* and 5*A*). An unexpected observation in *pga1-1* is the activation of genes involved in the control of proteostasis. In contrast to the repressed expression of *PhANGs*, transcript levels of *ctHSP70*, *cpHSP70*, and *HsfA2* were markedly increased in *pga1-1* (Fig. 7*A*). These genes are involved in protein quality control, and their upregulations were also observed in lincomycin-induced or *var2*-mediated cpUPR (15, 16). The upregulation of *ctHSP70* and *HsfA2* may represent a response to the stress caused by the overaccumulation of cytosol-translated chloroplast proteins in *pga1-1*. Compared with low levels of photosynthetic proteins, the relatively higher levels of Clp and cpHSP70 indicated an additional mechanism to maintain proteostasis in the chloroplast (Fig. 7). Taking advantage of the nonlethal *pga1-1* allele, we were able to show a synergistic interaction between *pga1-1* and *clpr1-2*, providing genetic evidence for the cooperation of AtFtsH12 and the Clp complex in regulating plant development. However, the molecular mechanism behind this synergistic interaction is still not clear. How these chaperon and protease systems cooperate in the cytosol and in the chloroplast to maintain proteostasis needs to be further explored.

Overall, it is possible that defects in chloroplast protein accumulation or chloroplast development in *pga1-1* are able to generate signals that repress the expression of both nuclear and chloroplast photosynthetic genes, while activating the expression of genes involved in maintaining protein homeostasis in both the cytosol and the chloroplast, implying a complex regulatory network. Future work with *pga1-1* and additional mutants and components in this network will shine more light on this critical process.

## Experimental procedures

### Plant materials and growth conditions

The WT *A. thaliana* and the *pga1-1* mutant are Columbia ecotype. The *pga1-1* mutant was identified from an ethyl-methanesulfonate mutagenesis pool and backcrossed with the WT for five times. The *ptac2-5 pTAC2-GFP* line is a complementation line of the T-DNA insertion mutant *ptac2-5* (SAIL_244_G05, CS811416) in the *pTAC2* gene, transformed with a *pTAC2-GFP* fusion gene driven by its native promoter (from our group’s unpublished data). The WT *p35S:FNR*_*ctp*_*-GFP* line is kindly provided by Dr Ralf Bernd Klösgen. After surface sterilization, Arabidopsis seeds were stratified at 4 °C for 2 days and grown on commercial soil mix (Pindstrup) or on half-strength Murashige and Skoog medium (1/2 MS) containing 1.0% Bacto Agar. The plant growth room was maintained at 22 °C under continuous light with a light intensity of ∼100 μmol photons m^−2^ s^−1^. In the climate chamber, plants were grown under 12-h day/12-h night cycle. For the de-etiolation assay, seeds were germinated on 1/2 MS for 3 days in the dark at 22 °C before transferring under light for 24 h.

### RNA manipulations and RT-qPCR

Total RNAs were extracted from 2-week-old seedlings or from de-etiolated seedlings grown on 1/2 MS, using the Trizol RNA reagent (Invitrogen) according to the manufacturer’s instruction. Reverse transcription reactions were performed with 1.0 μg total RNA using a PrimeScript Reverse Transcription Kit (Takara) and a mixture of oligo (dT)_18_ and random hexamer primers. RT-qPCR was carried out using the FastStart Essential DNA Green Master (Roche) on the CFX96 Real-Time PCR System (Bio-Rad). The relative transcript levels of each gene were analyzed using the comparative 2^−ΔΔCt^ method (57), and *ACT2* or *PP2A* were used as the reference gene. To ensure the reproducibility of RT-qPCR, three or four biological replicates were performed. All primers used in this study are listed in Table S1.

### Plasmid constructions and plant transformation

To complement *pga1-1*, two binary vectors, *pUBQ10:AtFtsH12* and *pAtFtsH12:gAtFtsH12-GFP*, were constructed. The full-length cDNA of *AtFtsH12* was amplified using primers *FtsH12 utrF* and *FtsH12 stcR* for *pUBQ10:AtFtsH12*. The full-length genomic DNA of *AtFtsH12* (*gAtFtsH12*) was amplified using primers *FtsH12F* and *FtsH12R* for *pAtFtsH12:gAtFtsH12-GFP*. PCR products were digested with *Bam*HI and cloned into a modified *pCambia1300* vector containing the *UBQ10* promoter and a *pCambia1300-GFP* vector, respectively. WT and homozygous *pga1-1* plants were transformed by the floral dip method (58). Transgenic lines were screened on 1/2 MS plates containing 25 mg l^−1^ hygromycin.

### Protein 3-D structures prediction

The PDB file of AtFtsH12 (alphafold.com/entry/Q9SAJ3) was obtained from the AlphaFold Protein Structure Database (59). The 3-D structure of ATP molecule (DB00171) was obtained from the DrugBank Online (60). The molecular docking between AtFtsH12 and ATP was performed with the AutoDock software (https://autodocksuite.scripps.edu/autodock4/) (61) and visualized with PyMOL 2.5.

### Preparation of anti-AtFtsH12 polyclonal antibodies

To express the loop domain of AtFtsH12, the cDNA fragment corresponding to the predicted loop region (amino acid residues 175–432) of AtFtsH12 was amplified using *FtsH12 gF* and *FtsH12 gR*, and cloned into *pET28a* with Gibson Assembly Cloning Kit (NEB), to generate *pET28a-AtFtsH12-Loop*. The recombinant AtFtsH12-Loop protein was expressed in *E. coli* BL21 (*DE3*) and purified with nickel-chelate affinity chromatography. The purified AtFtsH12-Loop protein was used as an antigen to produce anti-AtFtsH12 polyclonal antibodies.

### Yeast two-hybrid assays

The yeast two-hybrid assay was performed using the Matchmaker Gold Yeast Two-Hybrid System (Clontech). The coding regions for the loop domain (amino acid residues 175–432), the ATPase domain (amino acid residues 456–736), and the M41 domain (amino acid residues 756–961) of AtFtsH12 were cloned into the prey vector pGADT7. The coding region for the N-terminus of LhcB2 (amino acid residues 1–117) was cloned into the bait vector pGBKT7. The transformed clones were screened using plates containing double dropout SD medium lacking leucine and tryptophan and further using quadruple dropout SD medium lacking adenine, histidine, leucine, and tryptophan. The quadruple dropout SD medium plates supplemented with 20 mg l^−1^ X-α-Gal were used for high stringency.

### Chloroplast fractionation, total protein extraction, and immunoblotting

The chloroplast fractionation was performed as described (35), and the protein was loaded based on equal protein concentrations. Total protein extraction from 2-week-old leaves or de-etiolated seedlings was performed based on equal fresh tissue weight as described (29). For immunoblotting, protein samples were separated with 12% SDS-PAGE containing 8.0 M urea and transferred to PVDF membranes (0.22 μm, Millipore). Immunoblotting was performed with standard procedures, and signals were detected using Clarity Western ECL Substrate (Bio-Rad). The antibodies used in this study were listed in Table S2.

### Preparation of crude chloroplast membranes, soluble stromal proteins, and BN-PAGE

Preparation of crude chloroplast membranes and BN-PAGE were performed as described (29). Briefly, for 1-D BN-PAGE, crude membranes solubilized with the 25BTH20G buffer (25 mM Bis–Tris–HCl pH7.0 and 20% glycerol) containing 1% n-dodecyl-β-D-maltoside (w/v) or 2% digitonin (w/v) were resolved on 3 to 10% gradient native PAGE. As the chlorophyll content in *pga1-1* was reduced to ∼30% of that in the WT (Fig. 1*C*), the loading of thylakoids is based on the difference between their chlorophyll contents (corresponding to 6.0 μg chlorophylls from *pga1-1* and 20.0 μg chlorophylls from the WT sample). For 2-D SDS-PAGE, gel lanes were excised from the 1-D BN-PAGE and denatured in 2× SDS sample buffer at room temperature and was resolved on 12% SDS-PAGE containing 8.0 M urea. The AtFtsH12 complexes on 2-D gel were detected with immunoblotting using the AtFtsH12 antibodies. The PEP complex in *pga1-1* was analyzed with 3 to 10% BN-PAGE according to (62). For detecting Clp complexes, 2-week-old seedlings of WT and *pga1-1* were homogenized in liquid nitrogen. Total soluble proteins were extracted with the 25BTH20G buffer and resolved on 3 to 10% BN-PAGE gel.

### Confocal microscopy

To observe the localization of AtFtsH12-GFP, protoplasts were prepared from 4-week-old rosette leaves of *pga1-1 pAtFtsH12:gAtFtsH12-GFP* lines, using the enzyme solution (20 mM MES pH5.7, 0.4 M Mannitol, 20 mM KCl) containing 1.5% cellulase R10 (w/v) and 0.4% macerozyme R10 (w/v) to remove cell walls (63). Mesophyll protoplasts of *pga1-1 pAtFtsH12:gAtFtsH12-GFP* were collected directly by centrifugation at 100*g* for 2 min. To observe the fluorescence of pTAC2-GFP and FNR_ctp_-GFP, cotyledons or isolated protoplasts were used directly for confocal microscopy imaging. In order to compare fluorescent signal intensities in different genotypes, GFP or chlorophyll autofluorescence images were acquired with the same exposure time (for example, 600 milliseconds for GFP and 20 milliseconds for chlorophyll in Fig. S10). GFP fluorescence and chlorophyll autofluorescence were monitored with a spinning disk confocal microscope (Revolution-XD, Andor). Confocal images were processed and the chloroplast area, cell area, numbers of chloroplasts, and first pair of rosette leaf area were determined using ImageJ (https://imagej.nih.gov/ij/) (64).

### Statistical analyses

Scatter plots showed mean ± SD, including measurements of chlorophyll, RT-qPCR, and quantification of chloroplast area, cell area, numbers of chloroplasts, and first pair of rosette leaf area. Significance analyses were performed using a one-way or two-way ANOVA analysis, using GraphPad Prism 8 (https://www.graphpad.com/scientific-software/prism/).

## Data availability

All data presented are contained within the article.

## Supporting information

This article contains supporting information.

## Conflict of interest

The authors declare that they have no conflict of interest with the contents of this article.
