# Supplementary material for: Chloroplast envelope ATPase PGA1/AtFtsH12 is required for chloroplast protein accumulation and cytosol-chloroplast protein homeostasis in Arabidopsis
Source: J Biol Chem. 2022 Sep 14;298(10):102489. doi: 10.1016/j.jbc.2022.102489 (PMC9574505; doi:10.1016/j.jbc.2022.102489)
Supplement: Supplemental Figures S1–S11 and Table S1 [file mmc1.pdf]

Supporting Information

The following supplemental materials are available.

Fig. S1 The phenotype of *pga1-1*.

Fig. S2 The accumulation of photosynthetic complexes in *pga1-1*.

Fig. S3 Map-based cloning of the *PGA1* locus.

Fig. S4 Structural comparison of ATP binding pockets from wild type AtFtsH12 and mutant AtFtsH12^{G703R}.

Fig. S5 AtFtsH12 contains the conserved HExxH motif.

Fig. S6 The accumulation of AtFtsH12 in *pga1-1 pAtFtsH12:gAtFtsH12-GFP*.

Fig. S7 Overexpression of *AtFtsH12* can cause abnormal chloroplast and leaf development.

Fig. S8 The phenotype of *pga1-1*/+.

Fig. S9 Supporting information for Fig. 5A.

Fig. S10 The accumulation of pTAC2-GFP in *pga1-1*.

Fig. S11 The conserved loop of AtFtsH12 interacts with the N-terminus of LhcB2.

Table S1 Primers used in this study.

Table S2 Antibodies used in this study.

Fig. S1

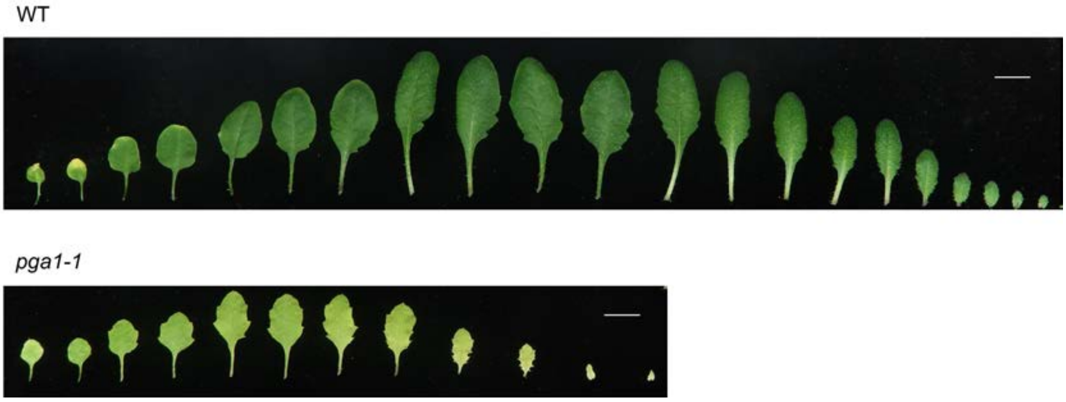

Figure S1. The phenotype of *pga1-1*. Rosette leaves of WT and *pga1-1* from Fig. 1B. Leaves were arranged in the order of initiation. Bars = 1.0 cm.

Fig. S2

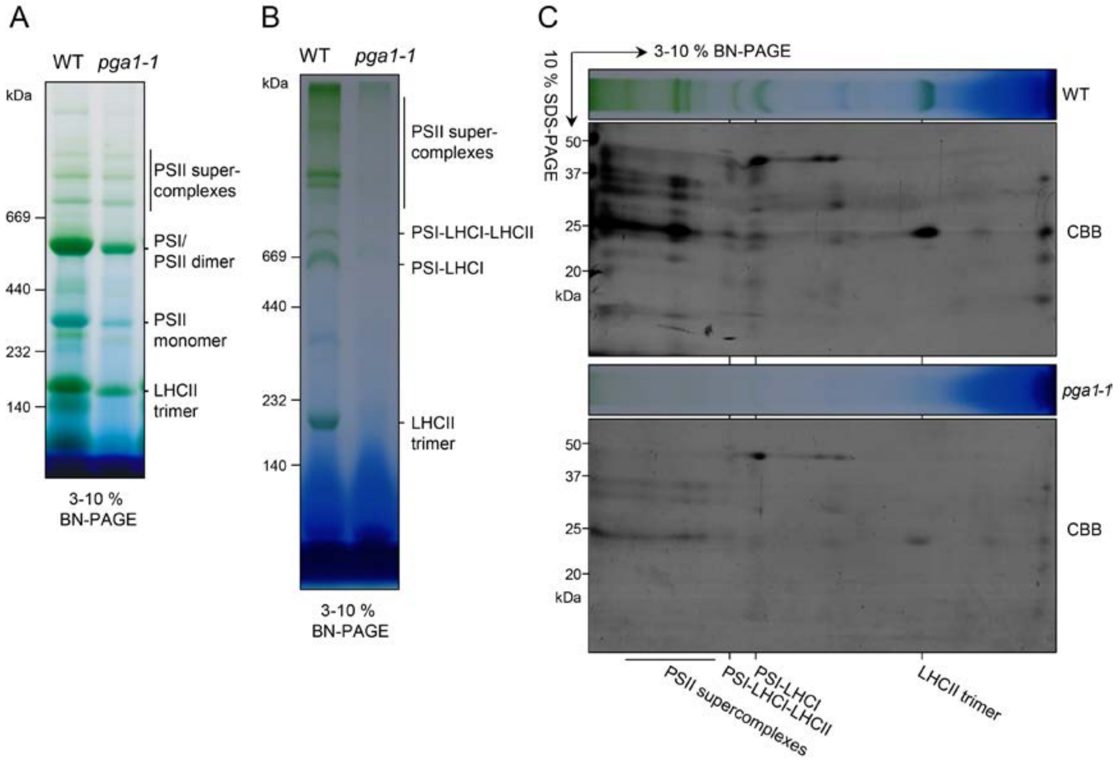

Figure S2. The accumulation of photosynthetic complexes in *pga1-1*. A-B. The 1-D BN-PAGE of WT and *pga1-1*. Thylakoid membranes were solubilized with 1 % β-DM (A) or 2 % digitonin (B). As the chlorophyll content in *pga1-1* was reduced to ~ 30 % of that in the WT (Fig. 1C), the loading of thylakoid protein is based on the difference between their chlorophyll contents (corresponding to 6.0 µg chlorophylls from *pga1-1* and 20.0 µg chlorophylls from the WT sample). C. The 2-D BN-PAGE analysis for the 1-D BN-PAGE in (B).

Fig. S3

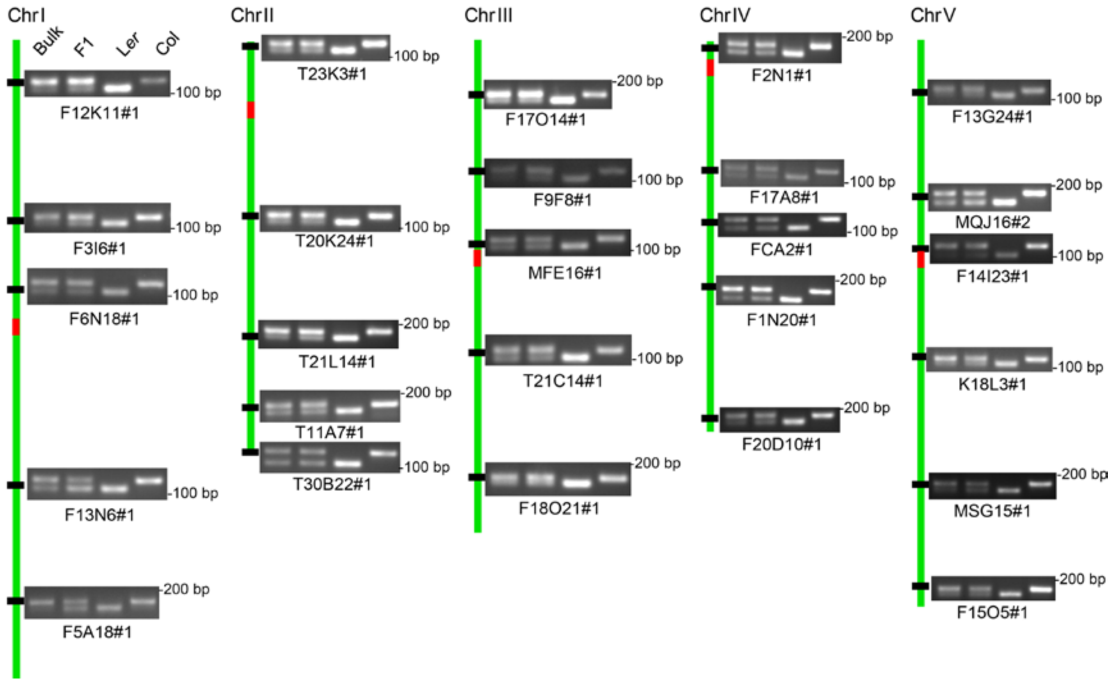

Figure S3. Map-based cloning of the *PGA1* locus. Bulked segregant analysis using mixed DNA pool from 95 individuals localized the *PGA1* locus to the chromosome 1, near the marker *F5A18#1*. For bulked segregant analysis, 25 markers that distributed on the five chromosomes of Arabidopsis were used.

Fig. S4

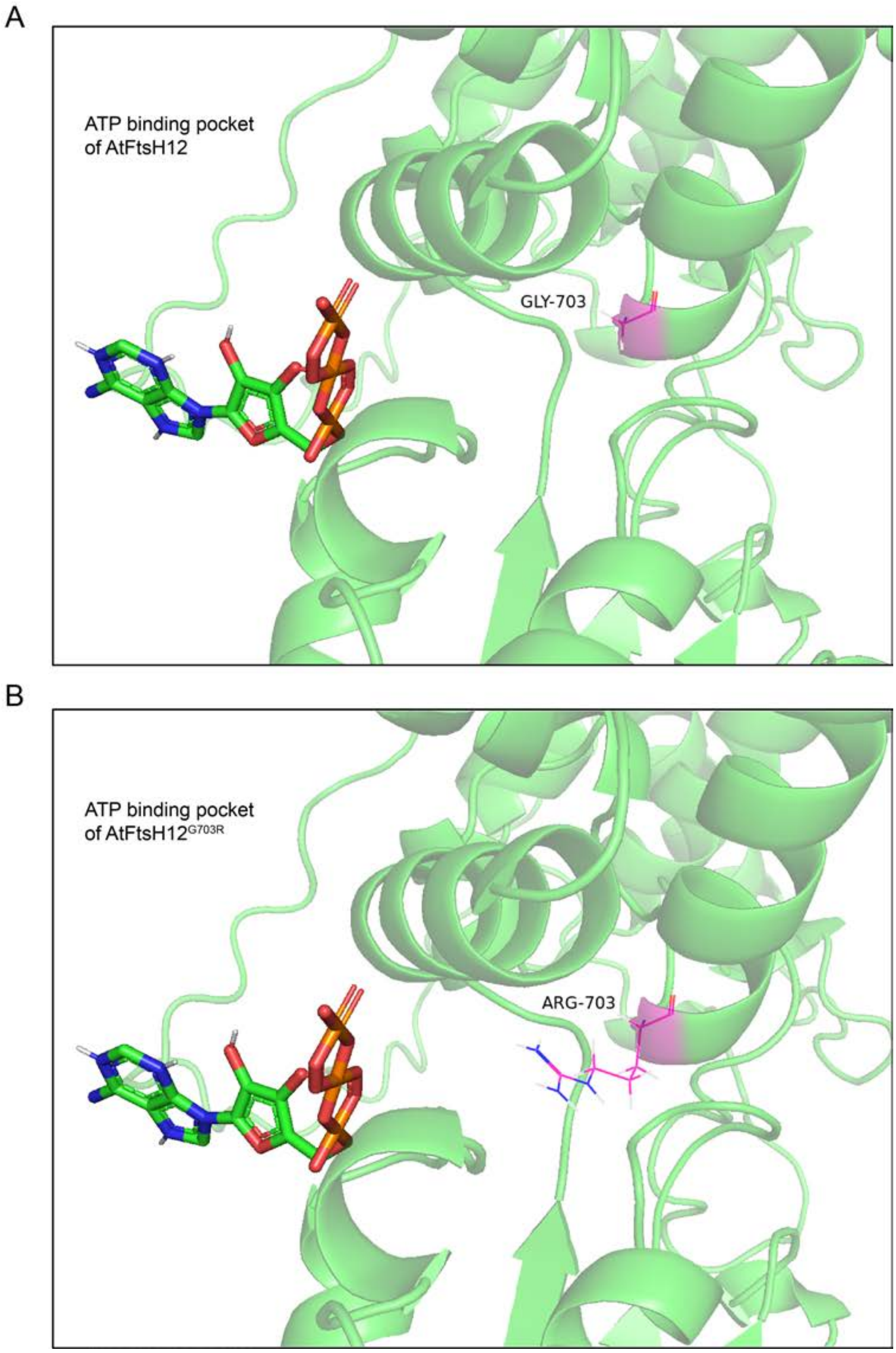

Figure S4. The structural comparison of ATP binding pockets from wild type AtFtsH12 (A) and mutant AtFtsH12^{G703R} (B). The structures of wild type and mutant forms of AtFtsH12 were obtained as described in Materials and Methods. The long side chain of Arginine 703 (R^{703}) in AtFtsH12^{G703R} extends into the ATP binding pocket.

Fig. S5

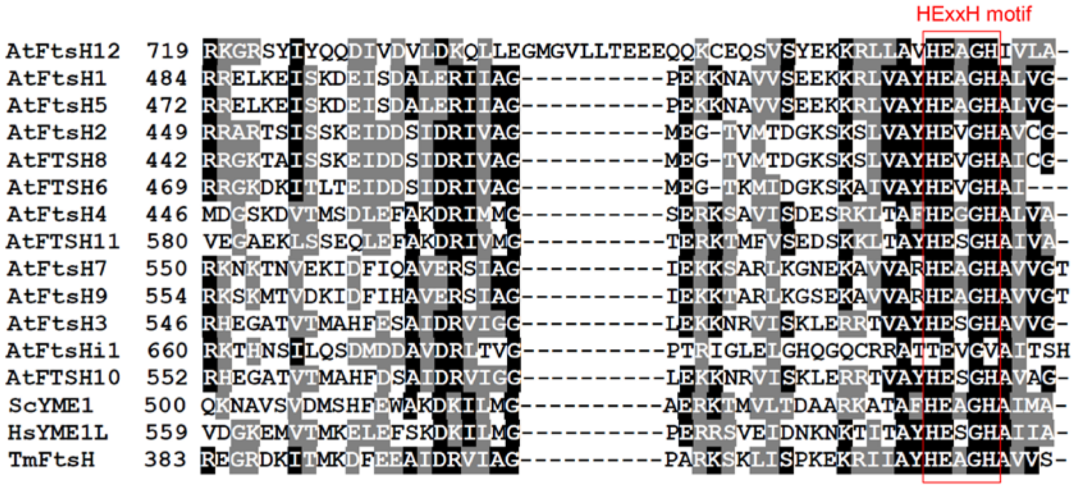

Figure S5. AtFtsH12 contains the conserved HExxH motif. AtFtsH homologs include 12 AtFtsHs from *Arabidopsis thaliana*, and ScYME1 from *Saccharomyces cerevisiae*, HsYME1L from *Homo sapiens*, and TmFtsH from *Thermotoga maritime*. The AtFtsHi1 belongs to the proteolytic FtsH inactive (FtsHi) due to mutations in the protease motif HExxH.

Fig. S6

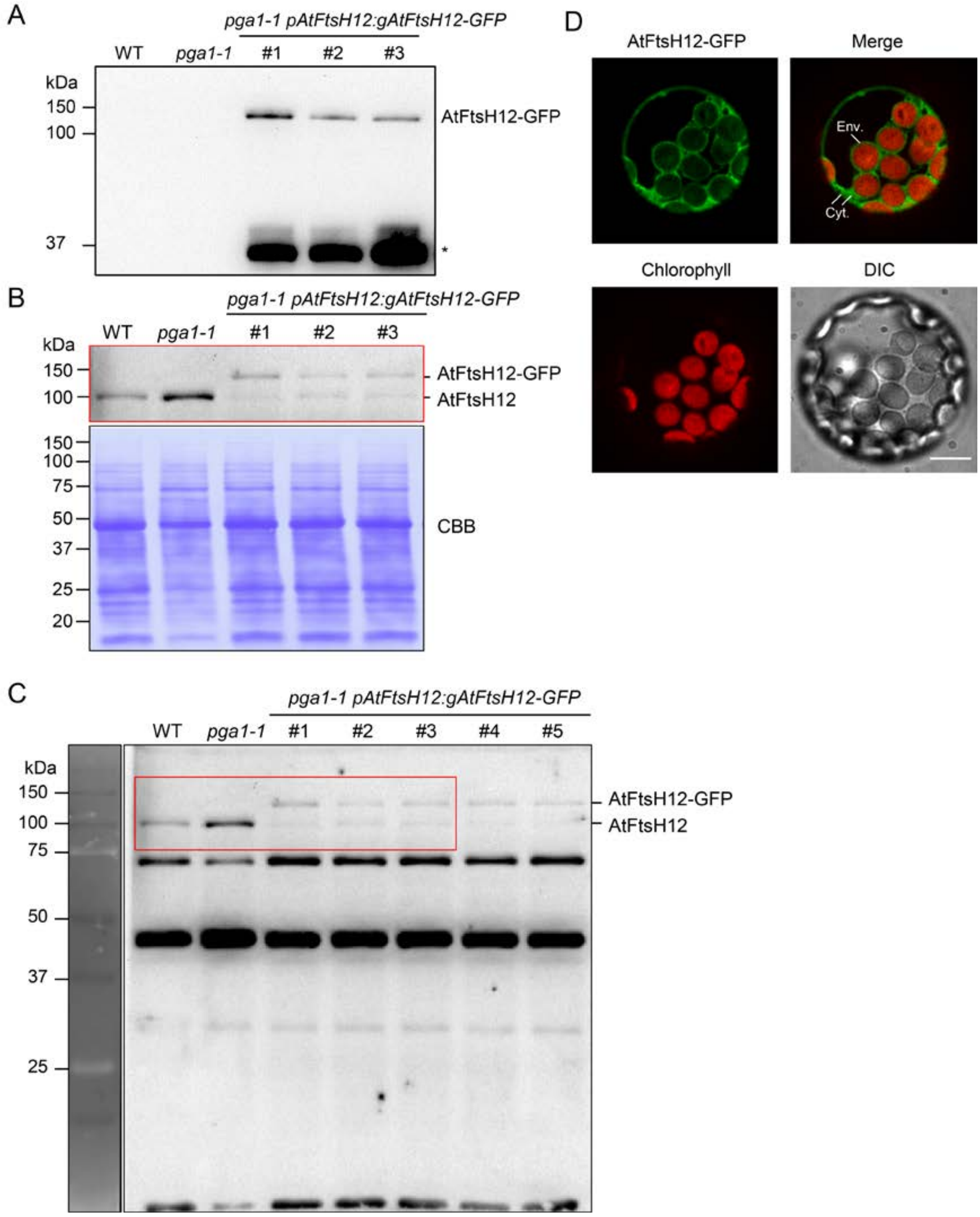

Figure S6. The accumulation of AtFtsH12 in *pga1-1 pAtFtsH12:gAtFtsH12-GFP*. A. The accumulation of AtFtsH12-GFP was detected using a GFP antibody. The asterisk indicates the degraded AtFtsH12-GFP. B. The accumulation of AtFtsH12-GFP and endogenous AtFtsH12 were detected using affinity purified AtFtsH12 polyclonal antibody. C. The original uncropped immunoblotting image for (B). The red box indicates the cropped area for (B). D. A replicate cell image to support Fig. 3A. Env for chloroplast envelope. Cyt for cytosol. Bar = 10 μm.

Fig. S7

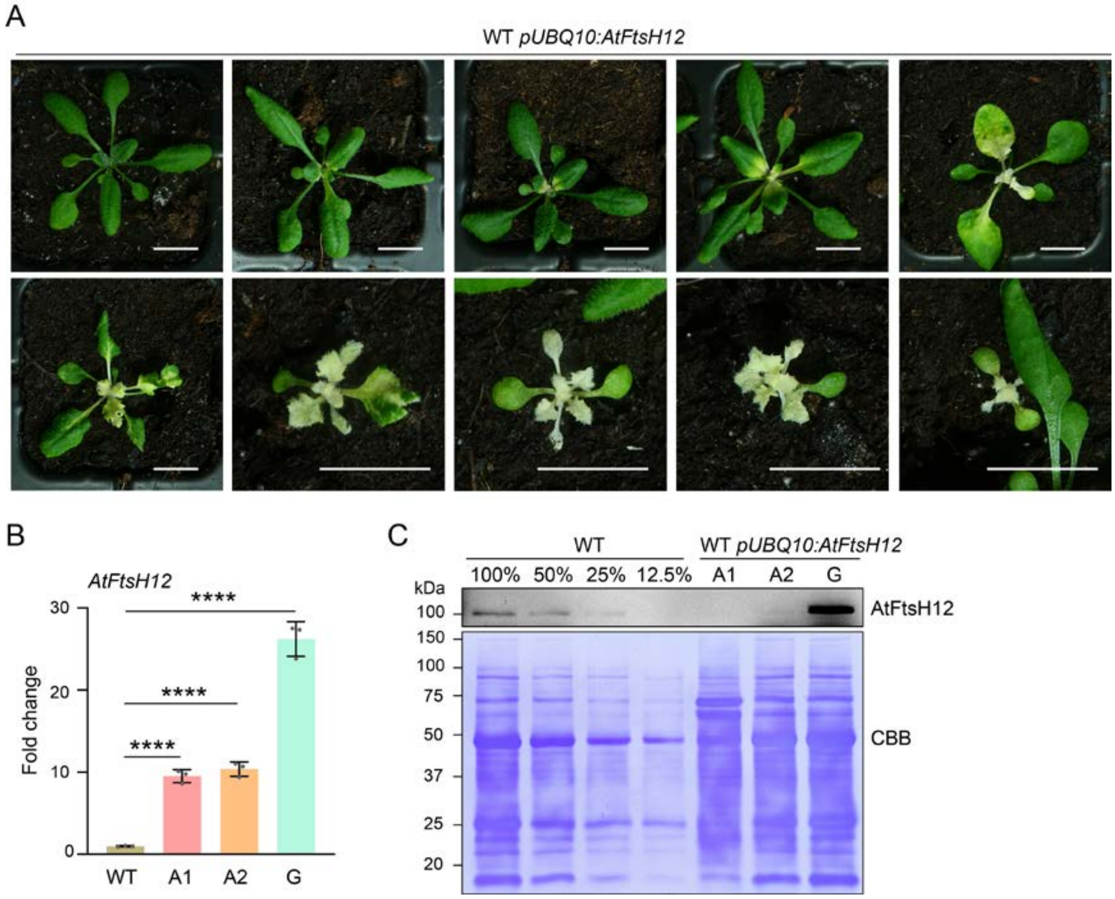

Figure S7. Overexpression of *AtFtsH12* can cause abnormal chloroplast and leaf development. A. Four-week-old WT *pUBQ10:AtFtsH12* T1 transgenic plants with different degrees of albino rosette leaves were shown. Bars = 1 cm. B. RT-qPCR analysis of the steady-state transcript levels of *AtFtsH12* in four-week-old WT and WT *pUBQ10:AtFtsH12* transgenic plants. Relative transcript levels with respect to those in the WT were calculated using the $2^{-\Delta\Delta Ct}$ method, and *PP2A* was used as the reference gene. Data are means ± s.d. of three biological replicates. **** p<0.0001. The A1 RNA or protein samples were isolated from albino leaves, the A2 samples from variegated leaves containing albino and green sectors, and the G samples from green leaves. C. The accumulation of AtFtsH12 protein in four-week-old WT and WT *pUBQ10:AtFtsH12* transgenic plants. Total proteins were extracted, and protein loading was normalized to equal fresh tissue weight and confirmed by the CBB-stained PVDF membrane.

Fig. S8

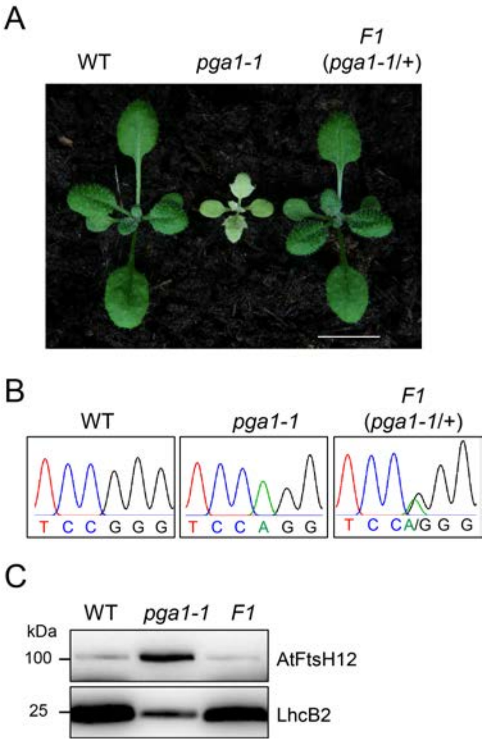

Figure S8. The phenotype of *pga1-1*/+. A. Representative two-week-old seedlings of WT, *pga1-1*, and *pga1-1*/+. Bar = 0.5 cm. B. DNA sequencing to confirm the genotypes of seedlings in (A). C. The accumulation of AtFtsH12 and LhcB2 in two-week-old WT, *pga1-1*, and *pga1-1*/+. Total proteins were extracted for immunoblotting using the anti-AtFtsH12 and anti-LhcB2 antibodies, and protein loading was normalized to equal fresh tissue weight.

Fig. S9

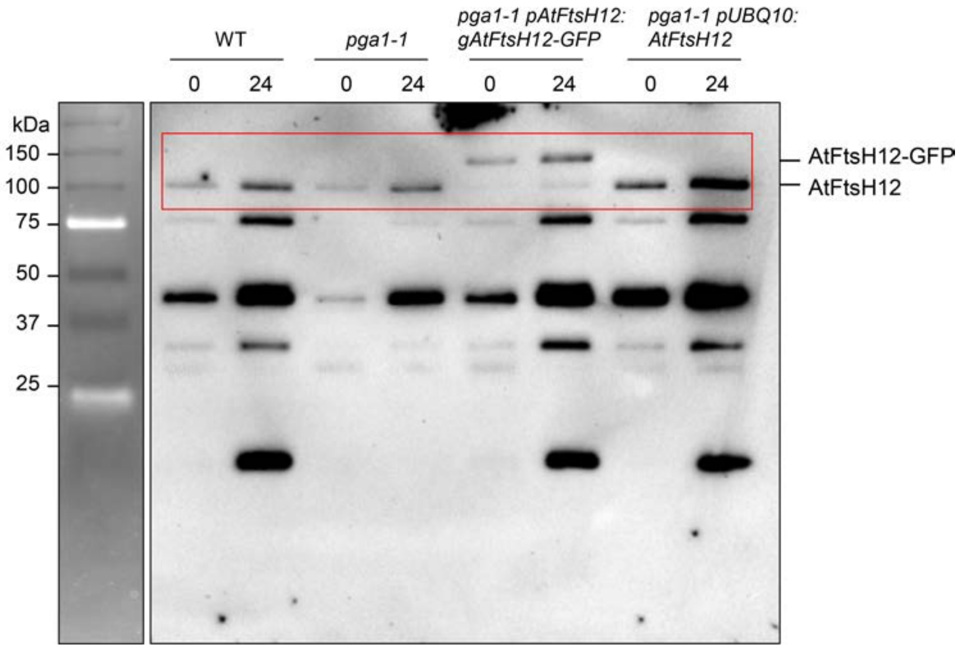

Figure S9. Supporting information for Fig. 5A. The original uncropped immunoblotting image of Figure 5A. Immunoblotting analysis was performed using unpurified AtFtsH12 antiserum. The red box indicates the cropped area for Figure 5A.

Fig. S10

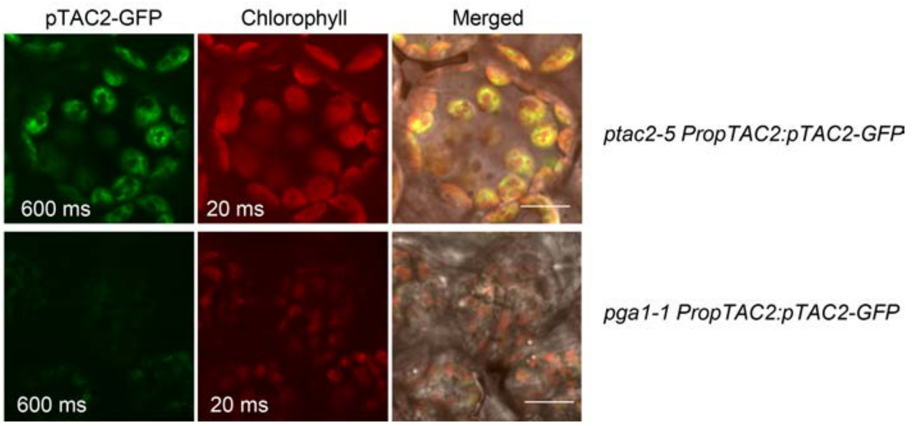

Figure S10. The accumulation of pTAC2-GFP in *pga1-1*. The accumulation of pTAC2-GFP in chloroplasts of 4-day-old *ptac2-5 PropTAC2:pTAC2-GFP* and *pga1-1 PropTAC2:pTAC2-GFP* were shown, respectively. Bars = 10 μm. In order to compare fluorescent signal intensities in different genotypes, GFP (600 milliseconds) or chlorophyll auto-fluorescence (20 milliseconds) images were acquired with the same exposure time.

Fig. S11

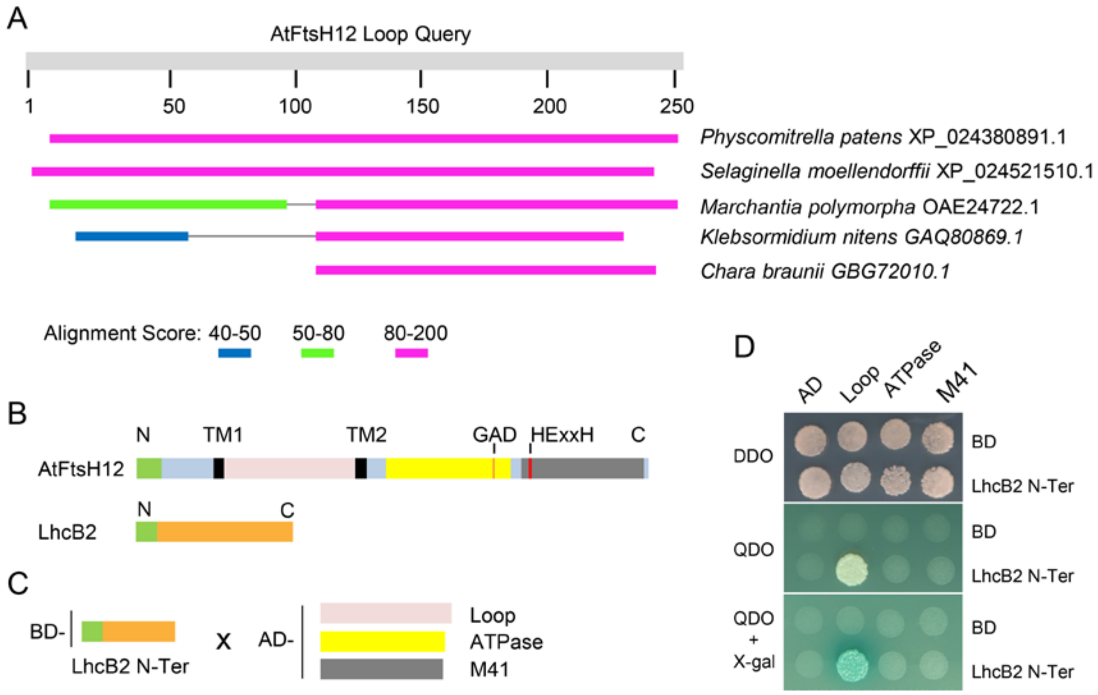

Figure S11. The conserved loop of AtFtsH12 interacts with the N-terminus of LhcB2. A. The conserved loop of AtFtsH12 homologs. BLASTP search was performed using the protein sequence of the AtFtsH12 loop as the query (258 amino acid residues), against protein database of *Klebsormidium nitens* (taxid:105231), *Chara braunii* (taxid:69332), *Marchantia polymorpha subsp. ruderalis* (taxid:1480154), *Physcomitrella patens* (taxid:3218), and *Selaginella moellendorffii* (taxid:88036). The partial alignment scores were indicated with colors. B-C. Schematics of domains of LhcB2 and AtFtsH12 used as bait and prey proteins in yeast two-hybrid assays. D. The loop region of AtFtsH12 interacted with the N-terminus of LhcB2 in yeasts. The QDO plates supplemented with X-α-Gal was used for high stringency.

Table S1. Primers used in this study

Primer name	Primer sequence (5’-3’)	Notes
FtsH12 utrF	CGCGGATCCCGAGTGAGCTGAATGGTTGGTACTC	*pUBQ10-AtFtsH12*
FtsH12 stcR	CGCGGATCCCTAGCTTCTGTGGAGTGGCGCAGCA	
FtsH12 F	CGCGGATCCGGCCCATTGAGAGTCAACCA	*pAtFtsH12-gAtFtsH12-GFP*
FtsH12 R	CGCGGATCCGCTTCTGTGGAGTGGCGCAG	
FtsH12-ATPase F	CCGGAATTCATGATCACATCCAAGCGCTTTCT	*pGADT7-AtFtsH12-ATPase*
FtsH12-ATPase R	CGCGGATCCTTATTTATCTAACACATCAACAATGTCTTGTTG	
FtsH12-loop F	CCGGAATTCATGCAAGCTCCTCGGGTAGAACG	*pGADT7-AtFtsH12-Loop*
FtsH12-loop R	CGCGGATCCTTATTTCATGAAGTAGTGGATCTGCCTCT	
FtsH12-M41 F	CCGGAATTCATGCAACAGAAATGTGAACAAAGTGTATCTTAC	*pGADT7-AtFtsH12-M41*
FtsH12-M41 R	CGCGGATCCTTAGCTTCTGTGGAGTGGCG	
cTP-LhcB2 F	CCGGAATTCATGGCCACATCAGCTATCCA	*pGBKT7-Lhcb2-Nter*
cTP-LhcB2 R	CGCGGATCCTTATAAGATGCTTTGCGCGTGGA	
FtsH12 gF	AAGAAGGAGATATACCATGGCACAAGCTCCTCGGGTAGAACG	*pET28a-AtFtsH12-Loop*
FtsH12 gR	TGGTGCTCGAGTGCGGCCGCTTTCATGAAGTAGTGGATCT	
79560 qF	TTAAGGATAGTGTGCACAAGG	*AtFtsH12* Real-Time-qPCR
79560 qR	AGAAAGTAACAGCGCAAAAGC	

FtsH12 qF1	AGCTGAATGGTTGGTACTCACCA	*AtFtsH12* 5’-UTR Real-Time-qPCR for Fig. S7B
FtsH12 qR1	CGATGAAGAGATAAGCGGATTCGG	
12580 qF1	AAATCATCGCCAACGATCAAG	*ctHSP70* Real-Time-qPCR
12580 qR1	GTATCTTCTTCCGATTAGACG	
26150 qF	AACATGGGTTTGCAGAATGTG	*HsfA2* Real-Time-qPCR
26150 qR	ATAGCTGCAACTTGACTCTTG	
24280 qF	AGGGTTTTTCAGCTTCAAACC	*cpHSP70* Real-Time-qPCR
24280 qR	GAAGAAGAAGAAGCGAATCCA	
LhcB2.2 3UTR qF	TGGCTGATCCTGTGGCTAACAAC	*LhcB2.2* Real-Time-qPCR
LhcB2.2 3UTR qR	AGTCCCAAGAAACATCATTAAGTGCCA	
psbA qPCR F	GGGAAGCTGCATCCGTTGAT	*psbA* Real-Time-qPCR
psbA qPCR R	CAGCTGCAACAGGAGCTGAAT	
RbcL qPCR F	GACAGGGAGTCAACTTTGGGC	*RbcL* Real-Time-qPCR
RbcL qPCR R	GATCTCGGTCAAAGCAGGCAT	
rpoB qF	TGTGTTAGCAGCTGCCGATCA	*rpoB* Real-Time-qPCR
rpoB qR	TGCTCCGGAGATAGTTCCCTTAACT	
pTAC2 qF	TCAATGAGGCAACAAAGCGTGG	*pTAC2* Real-Time-qPCR
pTAC2 qR	CAGCTAGTTGAGGAAGATCTCCCT	
AT2G24120 qF	ACCTTCCTATATCATGCGTACTCATGG	*rpoTp* Real-Time-qPCR
AT2G24120 qR	GTTGCCTCCATCAGCCCAGA	
PP2A qF	TATCGGATGACGATTCTTCGTGCAG	*PP2A* Real-Time-qPCR
PP2A qR	GCTTGGTCGACTATCGAATGAGAG	
ACT2 qF	CTTGCACCAAGCAGCATGAA	*Actin* Real-Time-qPCR
ACT2 qR	CCGATCCAGACACTGTACTTCCTT	
RbcS qPCR F	GCCACCCGCAAGGCTAACAA	*RbcS* Real-Time-qPCR
RbcS qPCR R	GGAATCCACTTGTTGCGGATAAGG	
ClpP1 qF	TGCGACCCGATGTACAGACAA	*ClpP1* Real-Time-qPCR
ClpP1 qR	AATTCTCCCGTTTGTGCCTCAT	
ClpP3 qF	AGGACTCAGAAAGAGACATTACGCT	*ClpP3*

ClpP3 qR	ACCAGAAGCAAGAAGAAACGCAC	Real-Time-qPCR
ClpR1 qF	GATAGCCCTGTCCGTAGGCAA	*ClpR1* Real-Time-qPCR
ClpR1 qR	CCACCTCCACCTCTGTACATCTG	

Table S2. Antibodies used in this study

Proteins	Antibodies (Catalog Number)
LhcA1	PhytoAB (PHY0043S)
AtFtsH12	Against loop region of AtFtsH12
LhcB2	Agrisera (AS01 003)
PsbA/D1	Agrisera (AS05 084)
VAR2/AtFtsH2	Qi et al., 2016
RbcL	Agrisera (AS03 037)
RbcS	Agrisera (AS07 259)
TOC34	Agrisera (AS07 238)
GFP	TaKaRa (632381)
rpoB	PhytoAB (PHY1700)
cpHSP70	PhytoAB (PHY0500)
ctHSP70	PhytoAB (PHY0167)
ClpP3	PhytoAB (PHY2069A)
